# GSK3B directs DNA repair choice and determines tumor response to PARP1 inhibition independent of BRCA1

**DOI:** 10.1172/JCI189956

**Published:** 2025-11-17

**Authors:** Heba S. Allam, Scarlett Acklin-Wehnert, Ratan Sadhukhan, Mousumi Patra, Fen Xia

**Affiliations:** 1 Department of Radiation Oncology, University of Arkansas for Medical Sciences, Little Rock, Arkansas, USA.; 2 Department of Radiation Oncology, Duke University Medical Center, Durham, North Carolina, USA.

**Keywords:** Cell biology, Oncology, DNA repair

## Abstract

Resistance to genotoxic therapies remains a major contributor to tumor recurrence and treatment failure, yet the mechanisms by which cancer cells escape these therapies through DNA damage response (DDR) activation are not fully understood. Here, we identify a DDR regulatory pathway in which glycogen synthase kinase 3 β (GSK3B), a multifunctional serine/threonine kinase, governs DNA double-strand break (DSB) repair pathway choice by phosphorylating 53BP1 at threonine 334 (T334) — a site distinct from canonical ATM targets. This phosphorylation event disrupts 53BP1’s interaction with nonhomologous end joining (NHEJ) effectors PTIP and RIF1, promoting their dissociation from DSBs and inhibiting 53BP1-driven NHEJ. Simultaneously, T334 phosphorylation facilitates the recruitment of CtIP and RPA32 for DNA end resection and promotes homologous recombination (HR) by enabling BRCA1 and RAD51 loading. Notably, the phospho-deficient T334A mutant of 53BP1, unlike 53BP1 loss, accumulates aberrantly at DSBs along with PTIP/RIF1, impairs end resection, and suppresses HR activity. Importantly, both genetic and pharmacologic disruption of the GSK3B–53BP1 axis sensitizes tumors to PARP inhibitors (PARPi) independently of BRCA1 status. Together, these findings reveal a GSK3B-dependent mechanism that regulates DSB repair pathway choice and provide a rationale for targeting this axis to enhance PARPi efficacy in solid tumors regardless of BRCA1 status.

## Abstract 2

GSK3B regulates DSB repair pathway choice by phosphorylating 53BP1 and provides a potential target to enhance PARPi efficacy in solid tumors regardless of BRCA1 status

## Introduction

Glycogen synthase kinase 3 β (GSK3B), a multifunctional serine/threonine kinase ubiquitously expressed in eukaryotic cells, has been implicated in numerous major processes including embryonic development, cell differentiation, apoptosis, and insulin response (1–5). Moreover, there is increasing evidence that aberrantly expressed GSK3B is involved in various cancer types and may promote cancer cell immortalization, proliferation, and invasion, while conferring resistance to radiation and chemotherapy agents (2, 6). In fact, multiple GSK3 inhibitors have been developed, including a GSK3B inhibitor currently being tested in a phase 1/2 trial for patients with advanced cancers (NCT03678883). Our previous studies have shown that inhibition of GSK3B alleviates cranial irradiation-induced cognitive toxicity in mice through protection against ionizing radiation–induced (IR-induced) apoptosis. GSK3B inhibition results in accelerated double strand break (DSB) repair efficiency in mouse hippocampal neurons. Specifically, inhibition of GSK3B enhances nonhomologous end joining (NHEJ) of DSBs (7, 8).

DNA damage response (DDR) is critical to the repair of lethal DSBs. A key rate limiting factor of NHEJ, p53 binding protein 1 (53BP1), dictates normal and tumor cell response to cancer treatment (9) and is required for immune cell class switch (10). NHEJ is one of the major DSB repair pathways, wherein 53BP1 assembles at DSB sites (11–13), stabilizes the DNA strands, and avoids nucleolytic degradation. Alternatively, homologous recombination (HR) is a complex and precise DNA repair process, which takes place mainly in S/G2 phase of cell cycle and relies on an undamaged template sister chromatid to resolve DSBs. 53BP1 plays a pivotal role in DSB repair pathway choice through promotion of NHEJ by suppression of DSB resection (12). Specifically, 53BP1 recruits 2 downstream effector proteins, RIF1 and PTIP, to DSB sites to compete with BRCA1-mediated HR in G1 phase (14). Conversely, BRCA1 removes 53BP1 from DSBs to allow resection and HR to proceed. The commitment to NHEJ versus HR has a marked impact on cell fate, genome integrity, carcinogenesis, and tumor treatment resistance. However, the factors and mechanisms determining the choice of DSB repair are still poorly understood.

Despite lacking enzymatic activity, 53BP1, as a critical DDR protein, contains interaction sites for numerous DSB-responsive proteins. Posttranslational regulation through ubiquitylation-mediated localization of 53BP1 to DSB sites or proteasome-mediated degradation plays an important role in the complex regulation of 53BP1 (15). Integral to its function are structural elements, including the BRCA1 carboxy-terminal (BRCT) repeats and 28 amino Ser/Thr-Gln (S/T-Q) sites located on the N terminus (9). The N terminus structure is important in maintaining DNA repair function in NHEJ. Phosphorylation of the N terminus by ATM promotes 53BP1’s recruitment to DSB sites and stabilization at these locations, inhibits end resection, enhances NHEJ activity, and promotes interaction with DNA damage–responsive proteins (9, 16, 17). Interestingly, dephosphorylation is also necessary for the dynamic recruitment of 53BP1 in the DDR. PP4 and PP2A are the main phosphatases dephosphorylating 53BP1 that tightly control 53BP1 for appropriate participation in the DDR and coordinate repair pathway choice (18). GSK3B has also been reported to crosstalk with 53BP1 (19). In contrast with our observation that GSK3B suppresses NHEJ activity in the repair of IR-induced DSBs in hippocampal neurons (7, 8), one study reported GSK3B phosphorylates 53BP1 at Ser166 and enhances DNA repair. Inhibiting GSK3B was shown to abolish 53BP1-mediated IR-induced DNA repair in cancer cells (20). Further investigation is needed to define the role and underlying mechanism of GSK3B in DSB repair.

In this study, we demonstrate that GSK3B binds and phosphorylates 53BP1 at threonine 334 amino acid (T334). Distinct from the role of ATM-mediated phosphorylation and promotion of 53BP1 in NHEJ activity, phosphorylation at T334 is critical to regulate 53BP1’s dynamic recruitment to DSB sites, manage the function of downstream mediators RIF1 and PTIP in their recruitment to and dissociation from DSBs, and control canonical NHEJ. In contrast, GSK3B-mediated phosphorylation of 53BP1 at T334 modulates the obstruction to CtIP and RPA32 access for DNA end resection and affects RAD51 recruitment, promoting HR repair. Most importantly, genetic and pharmacologic inhibition of the GSK3B-53BP1 signaling axis dramatically enhances the cytotoxic response of cancer in vitro and in vivo to PARP inhibitors (PARPi) independent of BRCA1 status.

## Results

### 53BP1 is a substrate of GSK3B.

**Figure 1:**
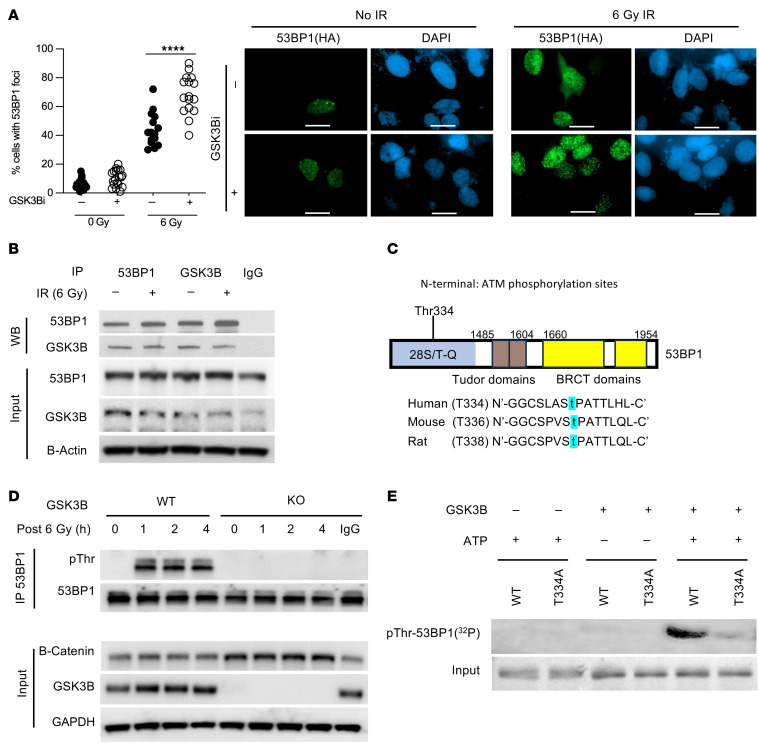
GSK3B phosphorylates 53BP1 at Threonine 334. (**A**) Representative images and quantification of 53BP1 foci-stained U2OS cells treated with GSK3Bi (30 μm lithium) before and after IR with 6Gy (*n* = 3). Scale bars:100 μm. (**B**) Coimmunoprecipitation assay of 53BP1 and GSK3B with or without IR (6 Gy). (**C**) The sequence of *53BP1*. Letters in blue mark the putative phosphorylatable threonine that matches the GSK3B consensus site. (**D**) In vivo phosphorylation assay by 53BP1 pulldown followed with antibody-mediated identification of phosphorylated threonine (pThr) in WT and *Gsk3b^–/–^* MEF cells before and after IR (6 Gy). (**E**) In vitro kinase assay in U2OS cells with coincubation of GSK3B, T334A 53BP1, WT 53BP1, and isotope-labeled phosphate with or without ATP. Statistical significance was assessed using a 1-way ANOVA with Tukey’s test. ****P *<* 0.0001.

Previous studies have shown that the role of GSK3B in regulation of DDR activity is controversial (7, 8, 20, 21). To define the underlying mechanisms by which GSK3B regulates DSB repair, we first examined the effect of GSK3B on the efficiency of DNA damage–induced nuclear foci formation of 53BP1. Using lithium, a classic inhibitor of GSK3B, to modulate GSK3B’s function in U2OS cells, we examined 53BP1 intracellular response to DNA damage–induced nuclear foci formation (Figure 1A). Even at baseline, we see a slight but statistically significant increase in 53BP1 foci formation with GSK3Bi (LiCl) compared with control. As expected, IR-induced DNA damage significantly increased nuclear foci formation compared with unirradiated cells. Importantly, lithium-mediated GSK3B inhibition resulted in a further 1.8-fold increase in nuclear foci formation. A similar effect was seen in HN33 cells, an immortalized cell line derived from the somatic cell fusion of mouse hippocampal neurons (Supplemental Figure 1A; supplemental material available online with this article; https://doi.org/10.1172/JCI189956DS1).

To study the underlying mechanism, we examined whether 53BP1 and GSK3B physically interact using reciprocal coimmunoprecipitation (co-IP). As demonstrated in Figure 1B, the coprecipitation of endogenous 53BP1 with GSK3B was observed at basal level and increased in response to IR, indicating a physical interaction between the 2 proteins. To determine whether 53BP1 is a substrate of GSK3B kinase, we performed bioinformatic analysis of the 53BP1 amino acid sequence to identify a potential GSK3B phosphorylation site. Consensus sequences were identified within 53BP1 at T334 located within the previously described 28 N-terminal amino acids (Figure 1C). We next examined whether DNA damage induces GSK3B-dependent threonine phosphorylation of 53BP1 using mouse embryonic fibroblast (MEF) cells from WT or *Gsk3b^–/–^* mice and challenged them with 6 Gy IR. The phosphorylation of 53BP1 was analyzed by pull down of endogenous 53BP1 and identification of phosphorylated threonine (pThr) using a phospho-threonine-specific antibody. As shown in Figure 1D, we observed a marked increase in 53BP1 pThr in WT cells 1 hour after IR exposure. In contrast, we detected no pThr in *Gsk3b^–/–^* cells in response to IR despite equivalent 53BP1 pulldown. These results strongly indicate that DNA-damage–induced threonine phosphorylation of 53BP1 is dependent on GSK3B. These findings were validated in vivo utilizing siRNA-mediated *GSK3B* inhibition in U2OS cells (data not shown).

To verify this finding, we performed an in vitro GSK3B kinase assay using purified GSK3B and recombinant GSK-53BP1 fragments encompassing the entire length of 53BP1. Strong GSK3B-dependent phosphorylation of 53BP1 was observed only in the N-terminal fragment (53BP1-N1 [aa 1–361]), confirming the N1 fragment that contains the consensus motif was phosphorylated directly by GSK3B. Furthermore, GSK3B inhibition eliminated phosphorylation of 53BP1 (Supplemental Figure 1B). To confirm that GSK3B-dependent 53BP1 phosphorylation occurs at T334, we repeated the in vitro kinase assay with a Thr to Ala at aa334 mutant 53BP1 (T334A). Compared with WT 53BP1, which was phosphorylated in a GSK3B- and ATP-dependent manner, diminished phosphorylation was observed with T334A 53BP1, confirming T334 as a target residue for phosphorylation (Figure 1E). Together, these data confirm GSK3B phosphorylates 53BP1 at T334.

### GSK3B-mediated phosphorylation of 53BP1 on T334 regulates 53BP1 function in NHEJ.

**Figure 2:**
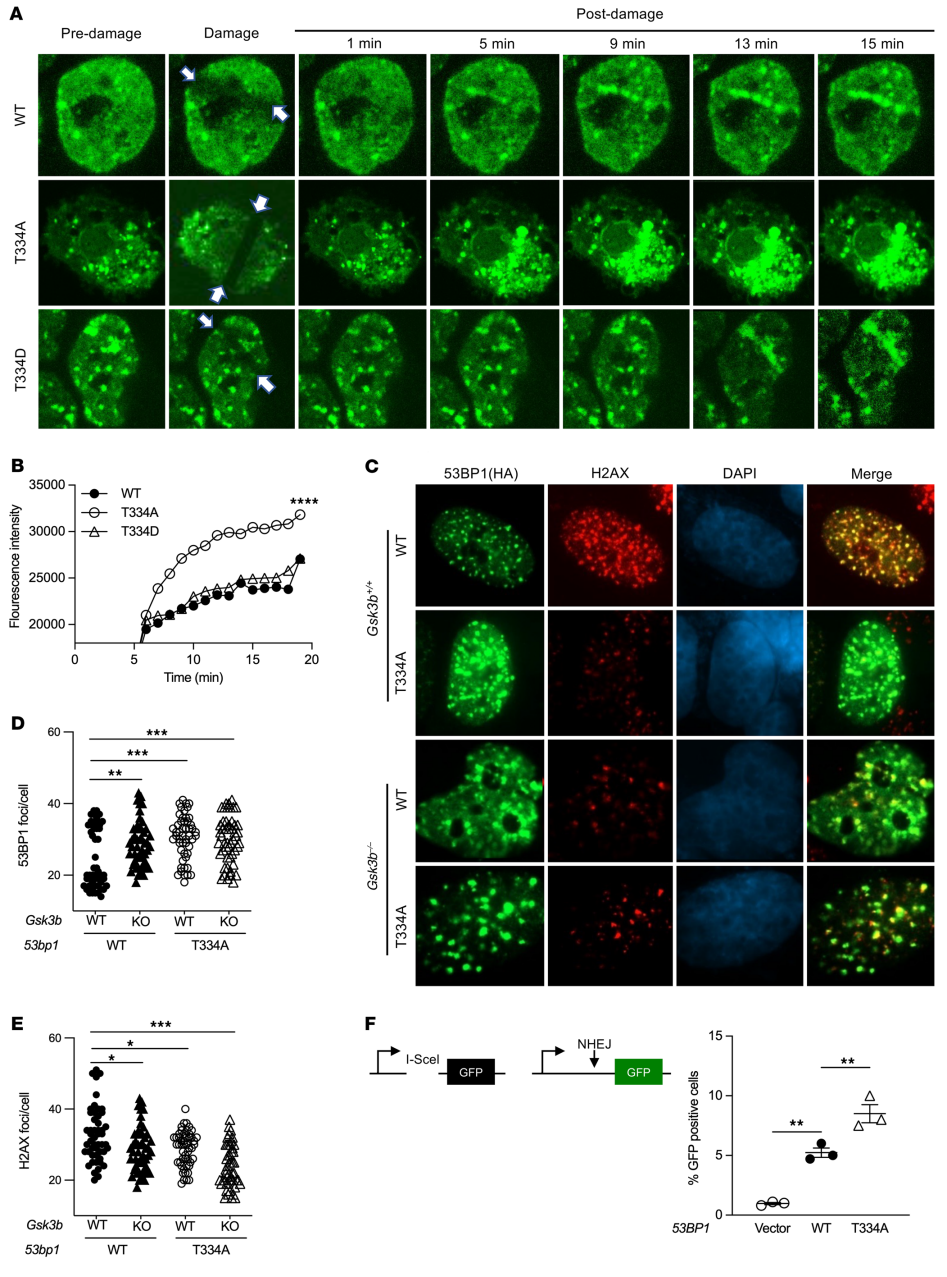
Effects of GSK3B-mediated phosphorylation at T334 on 53BP1 functions in non-homologous end-joining (NHEJ) repair of DSBs. (**A**) Recruitment kinetic analysis of 53BP1 live cell imaging. *53BP1^–/–^* U2OS cells reconstituted with the indicated GFP-tagged 53BP1 were damaged with microbeam laser and analyzed at the indicated time points by confocal microscopy. White arrows indicate the laser path. Magnification ×100. (**B**) Quantification of fluorescence intensity of microirradiated U2OS cells reconstituted with the indicated *53BP1*. (**C**) Representative images of 53BP1 and H2AX foci in WT or *Gsk3b^–/–^* MEF cells expressed with HA-tagged WT or T334A 53BP1. Magnification ×65. (**D**) 53BP1 foci per MEF cell (*n* = 3). (**E**) H2AX foci per MEF cell (*n* = 3). (**F**) Schematic of I-SceI NHEJ reporter assay. Assessment of NHEJ repair of DSBs as measured by percentage GFP-positive *53BP1^–/–^* U2OS cells reconstituted with various 53BP1 alleles. *n* = 3. Statistical significance was assessed using 1- and 2-way ANOVA with Tukey’s test. *P *<* 0.05; **P < 0.01; ***P < 0.001; ****P < 0.0001.

To determine the biological roles of the T334 site, we next asked if phosphorylation of T334 regulates the function of 53BP1 in DNA repair. *53BP1* was knocked out in U2OS cells using CRISPR followed by stable reconstitution with GFP-WT, -phosphorylation–deficient T334A mutant, and -phosphomimic T334D mutant. DNA damage was induced in U2OS cells using laser-induced microirradiation (path indicated by white arrow in figure), and different alleles of 53BP1 recruitment to sites of DSBs were visually quantified by GFP-53BP1 intensity (22) (Figure 2A). WT 53BP1 was promptly recruited to sites of DSBs within minutes of microirradiation with increasing levels at subsequent time points. Notably, recruitment and retention of 53BP1 at DSB sites were significantly increased in the T334A mutant when compared with WT 53BP1. In contrast, phosphomimic T334D demonstrated similar activity of recruitment and retention as WT. This significant increase in recruitment and retention of T334A remained through 20 minutes at the end of the experiment (Figure 2B).

Next, we assessed DNA-damage–induced 53BP1 nuclear foci formation in MEF cells from WT or *Gsk3b^–/–^* mouse, in which alleles of exogenous HA-53BP1 were stably expressed (Figure 2C and Supplemental Figure 2A). 53BP1 nuclear foci formation was scored by HA-positive ICC staining. Compared with WT, we found that loss of T334 phosphorylation by T334A mutation or knock out of *Gsk3b* resulted in a significant increase in 53BP1 nuclear foci formation at 1 hour following 6 Gy of IR. Cells with combined *Gsk3b* knockout and T334A mutation showed similar levels of increase in 53BP1 foci formation, suggesting that GSK3B and 53BP1 function in the same DDR pathway (Figure 2D). Together, this suggests that GSK3B suppresses 53BP1 recruitment to DNA damage sites through phosphorylation of T334.

Corresponding with the increased 53BP1 function, inhibition of T334 phosphorylation by T334A mutation or *Gsk3b* knockout resulted in reduced residual DSBs as indicated by decreased gH2AX foci in MEF cells (Figure 2E). Importantly, the combination of *53bp1* mutation and *Gsk3b* loss did not further decrease H2AX foci formation. These results were validated in U2OS cells (Supplemental Figure 2, B–E). 53BP1 nuclear foci formation increased with mutation of T334A *53bp1* compared with WT. Pharmacologic inhibition of GSK3B also increased nuclear foci formation, but combined inhibition of GSK3B and *53BP1* T334A mutation did not further increase 53BP1 nuclear foci formation in U2OS cells (Supplemental Figure 2C). H2AX foci formation again decreased with mutation of *53BP1* or loss of GSK3B function (Supplemental Figure 2D).

To further determine the effect of T334 phosphorylation on DSB repair at the chromosomal level, we examined the efficiency of canonical NHEJ–mediated (c-NHEJ–mediated) repair of restriction enzyme I-SceI–induced DSBs utilizing a chromosomal c-NHEJ–specific reporter in which GFP transcription (23) requires c-NHEJ repair of DSBs (Figure 2F). *53BP1^–/–^* U2OS cells carrying the c-NHEJ reporter were reconstituted with different *53BP1* alleles, and c-NHEJ repair efficiency was assessed by quantifying the percentage of GFP-positive cells. Activity of NHEJ repair diminished in cells with *53BP1^–/–^* and was restored in cells with reconstitution of WT *53BP1* expression. Again, T334A mutant expression demonstrated further significantly increased NHEJ activity compared with WT (Figure 2F and Supplemental Figure 2F).

**Figure 3:**
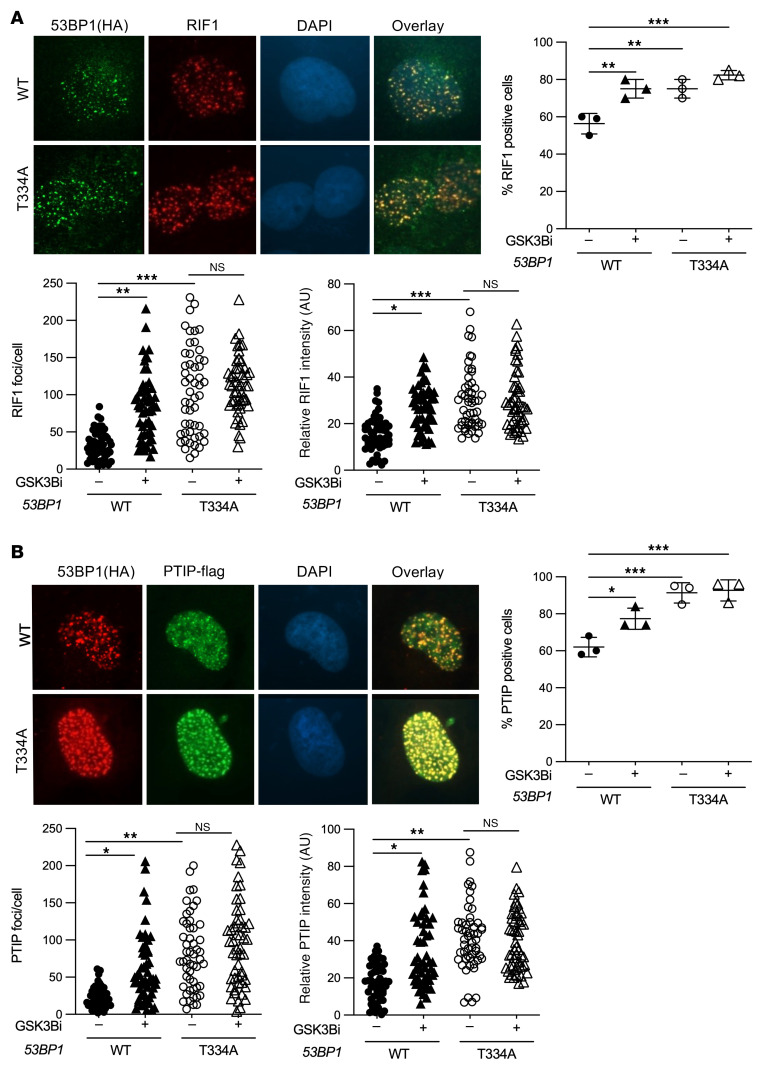
Effects of GSK3B-mediated phosphorylation at T334 on 53BP1 functions in NHEJ repair of DSBs cont. (**A**) Representative images of *53BP1^–/–^* U2OS cells reconstituted with HA-tagged WT or T334A 53BP1 and irradiated with 6Gy IR to induce DNA damage. Cells were stained for 53BP1(HA) and RIF1. Percentage of U2OS cells with or without GSK3B inhibitor that were positive for RIF1. *n* = 3. Absolute number of RIF1 foci per U2OS cell with or without GSK3B inhibitor. *n* = 3. Relative RIF1 foci intensity in U2OS cells with or without GSK3B inhibitor. *n* = 3. (**B**) Representative images of *53BP1^–/–^* U2OS cells reconstituted with HA-tagged WT or T334A 53BP1 and irradiated with 6 Gy to induce DNA damage. Cells were stained for 53BP1(HA) and PTIP. Percentage of U2OS cells with or without GSK3B inhibitor that were positive for PTIP. *n* = 3. Absolute number of PTIP foci per U2OS cell with or without GSK3B inhibitor. *n* = 3. Relative PTIP foci intensity in U2OS cells with or without GSK3B inhibitor. *n* = 3. Relative intensity was calculated for each cell by the Image J software. Statistical significance was assessed using 1- and 2-way ANOVA with Tukey’s test. *P < 0.05; **P < 0.01; ***P < 0.001. Magnification ×65.

After observing that T334 phosphorylation affects NHEJ function, we examined whether recruitment of RIF1 and PTIP, well-documented NHEJ mediators, to DNA damage sites would also be affected. U2OS cells with genetically manipulated T334 were stained for RIF1 and PTIP to assess IR-induced nuclear foci formation (Figure 3, A and B). Compared with cells with normal T334 phosphorylation, inhibition of T334 phosphorylation genetically with T334A mutation and pharmacologically with GSK3B inhibitor (SB216763) significantly increased the percent of cells with RIF1 (Figure 3A) and PTIP foci (Figure 3B). GSK3B inhibition had no further effect on RIF1 and PTIP foci in cells expressing T334A mutant. Consistent effects of GSK3B-dependent T334 phosphorylation on RIF1 and PTIP functions were observed when the number per cell and intensity of foci were analyzed (Figure 3, A and B). These data demonstrate GSK3B-mediated phosphorylation at T334 also leads to suppression of RIF1 and PTIP function in 53BP1-dependent recruitment to DSB sites. Together, these data strongly suggest that GSK3B controls 53BP1 function in NHEJ through phosphorylation of T334 and results in decreased recruitment and retention at DSB sites, reduced 53BP1 nuclear foci formation, and suppressed c-NHEJ activity.

### Phosphorylation of 53BP1 at threonine 334 modulates its recruitment kinetics to DNA damage sites following ionizing radiation.

**Figure 4:**
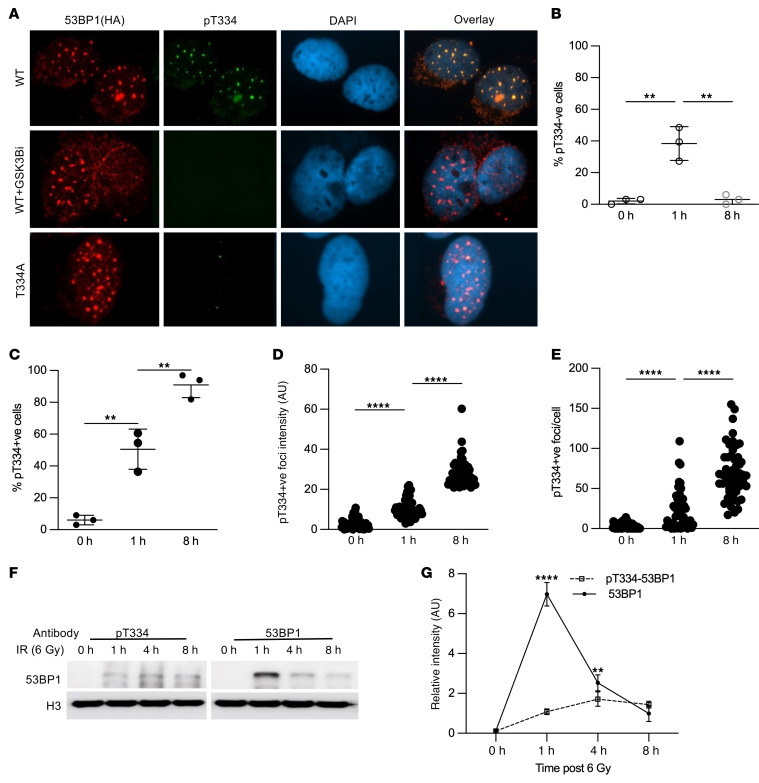
Phosphorylation of 53BP1 at threonine 334 modulates its recruitment kinetics to DNA damage sites following ionizing radiation. (**A**) Representative images of *53BP1^–/–^* U2OS cells reconstituted with HA-tagged WT or T334A 53BP1 or WT treated with GSK3B inhibitor and irradiated with 6 Gy to induce DNA damage. Cells were stained for T334 phospho-specific antibody. Magnification ×65. Quantification of (**B**) percentage pT334A-ve cells or (**C**) pT334A+ve cells at different time points following radiation. *n* = 3. (**D**) Relative intensity and (**E**) number of foci per cell were assessed for each cell by the Image J software. *n* = 3. (**F**) Western blot analysis of the chromatin binding kinetics of T334 phosphorylated 53BP1 (left panel) versus total 53BP1 by probe with phospho-T334 specific antibody or regular 53BP1 antibody. (**G**) Quantification of 53BP1 and pT334 53BP1 bound to chromatin. Data were analyzed by 1- and 2-way ANOVA followed by Tukey’s multiple comparison test. Values are mean ± SEM. **P < 0.01; ****P < 0.0001.

To further characterize the role of T334 in 53BP1’s function, we generated a specific antibody recognizing T334’s phosphorylation. The specificity of the antibody was validated on Western blot and ICC in *53BP1^–/–^* U2OS cells reconstituted with HA-tagged WT or T334A 53BP1 (Figure 4A) and with Western blot in U2OS (Supplemental Figure 3A) and *Gsk3b^+/+^* and *Gsk3b^–/–^* MEF cells (Supplemental Figure 3B). The T334 phospho-specific antibody does not recognize the T334A mutant and stained negative when cells were treated with the GSK3B inhibitor SB216763. The kinetics of DNA damage–induced nuclear foci of phospho-T334 (pT334+ve) versus nonphosphorylated T334 (pT334-ve), as defined by HA-positive and T334 phospho-specific antibody negative staining, were then examined following IR. We observed that pT334-ve foci increased quickly at 1 hour and returned to basal level late at 8 hours after 6 Gy IR (Figure 4B), consistent with reports by many studies. In contrast, pT334+ve foci formation increased at 1 hour following DNA damage and continued to increase significantly at 8 hours (Figure 4C). The analysis of pT334+ve foci intensity (Figure 4D) and number/cell (Figure 4E) again demonstrated similar kinetics where they increased dramatically more than 1-fold at the 8-hour late time point following DNA damage. This is distinct from c-NHEJ kinetics but mimics HR repair kinetics. We further studied whether T334 phosphorylation affected chromatin binding by 53BP1. WT U2OS cells were irradiated with 6 Gy to induce DNA damage and submitted for chromatin Western blot by probing with pT334 or regular 53BP1 at various time points (Figure 4F). As shown in Figure 4, F and G, 53BP1 binds quickly at 1 hour and evicts quickly but retains at a level significantly higher than basal level at 8 hours. In contrast, phosphorylated T334 binds significantly less at 1 hour with slow increase through 8 hours (Figure 4, F and G). Interestingly, chromatin-bound total 53BP1 and T334 phosphorylated 53BP1 reached the same level, indicating that chromatin-bound 53BP1 at 8 hours is approximately majority T334 phosphorylated.

### GSK3B-mediated phosphorylation of 53BP1 at T334 promotes ssDNA resection and HR-mediated DSB repair.

53BP1 binds to DSB sites, promotes NHEJ repair, and blocks the DSB end from being accessed by CtIP, thereby suppressing single strand DNA resection, which initiates HR-mediated DSB repair. The high level of pT334 foci at late phase following DNA damage leads us to hypothesize that phosphorylation at T334 may confer functions distinct from 53BP1’s canonical role in promoting NHEJ and may be involved in HR activity. As an early, integral initiation process in HR, CtBP-interacting protein (CtIP) recognizes and processes DSB sites (24) along with the MRN complex (25), generating single strand DNA (ssDNA) at the DSB ends that is quickly bound by RPA protein (26). RPA is then replaced by RAD51, facilitated by the breast cancer susceptibility protein (BRCA1), and organizes strand invasion and HR (27).

**Figure 5:**
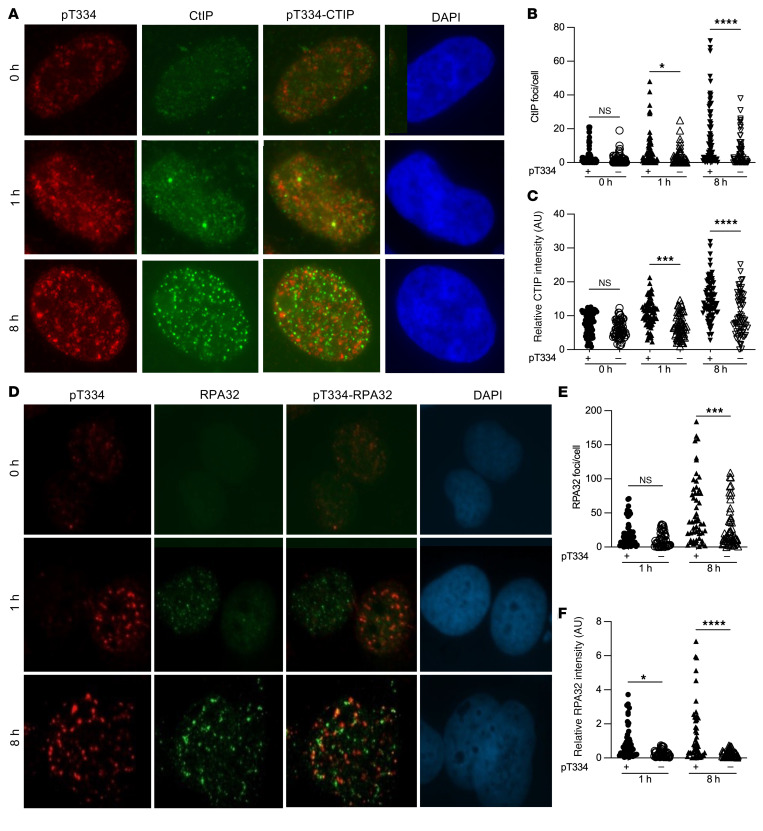
T334 phosphorylation of 53BP1 promotes HR repair of DSBs. (**A**) Representative images of *53BP1^–/–^* U2OS cells reconstituted with HA-tagged WT 53BP1 and irradiated with 6 Gy. Cells were costained for CtIP and p-T334 phospho-specific antibodies. (**B**) Number of CtIP foci per cell stratified by pT334 status at different time points following radiation (*n* = 3) and (**C**) relative intensity of CtIP foci at various time points. (**D**) Representative images of *53BP1^–/–^* U2OS cells reconstituted with HA-tagged WT 53BP1 and irradiated with 6 Gy. Cells were costained for RPA32 and p-T334 phospho-specific antibodies. (**E**) Number of RPA32 foci per cell stratified by pT334 status at different time points following radiation (*n* = 3) and (**F**) relative intensity of RPA32 foci at various time points. Data were analyzed by 2-way ANOVA followed by Tukey’s multiple comparison. Values are mean ± SEM. *P < 0.05; ***P < 0.001; ****P < 0.0001. Magnification ×65 (**A** and **D**).

**Figure 6:**
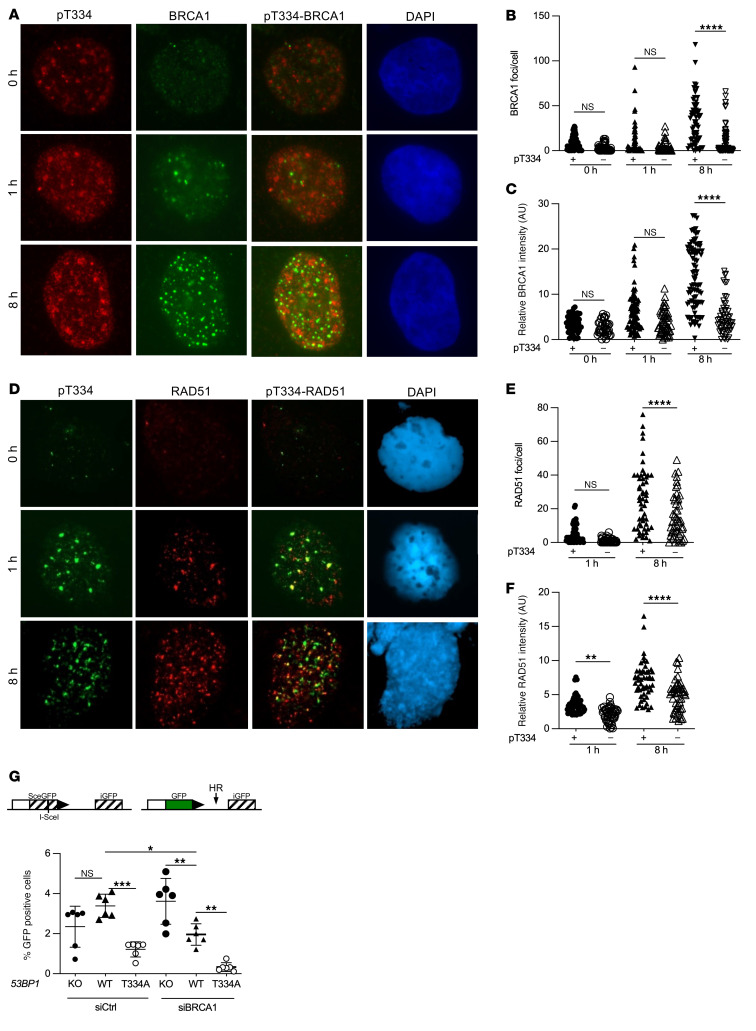
T334 phosphorylation of 53BP1 promotes HR repair of DSBs cont. (**A**) Representative images of *53BP1^–/–^* U2OS cells reconstituted with HA-tagged WT 53BP1 and irradiated with 6 Gy. Cells were costained for BRCA1 and p-T334 phospho-specific antibodies. (**B**) Number of BRCA1 foci per cell stratified by pT334 status at different time points following radiation and (**C**) relative intensity of BRCA1 foci at various time points. (**D**) Representative images of *53BP1^–/–^* U2OS cells reconstituted with HA-tagged WT 53BP1 and irradiated with 6 Gy. Cells were costained for RAD51 and T334 phospho-specific antibodies. (**E**) Number of RAD51 foci per cell stratified by pT334 status at different time points following radiation and (**F**) relative intensity of RAD51 foci at various time points. Quantification of foci and intensity were calculated for each cell by the Image J software. *n* = 3. (**G**) Schematic of I-SceI HR reporter assay. Assessment of chromosomal HR repair of DSB as measured by percentage GFP-positive *53BP1^–/–^* U2OS cells reconstituted with various 53BP1 alleles with or without *BRCA1* knockdown. DSBs were induced by adenoviral delivery of I-SceI, and the ability of cells to resolve DSBs by HR was assessed 48 hours later. *n* = 3. Data were analyzed by 2-way ANOVA followed by Tukey’s multiple comparison. Values are mean ± SEM. *P < 0.05; **P < 0.01; ***P < 0.001; ****P < 0.0001. Magnification ×65 (**A** and **D**).

To investigate the role of T334 in HR, we measured the formation of CtIP foci in 53BP1 modified U2OS cells following irradiation by costaining pT334 with CtIP (Figure 5A). We show that CtIP increased at 1 hour and continued to increase at 8 hours. Interestingly, CtIP was significantly increased in pT334+ve cells compared with pT334-ve cells (Figure 5, A–C, and Supplemental Figure 4A). Together, these data support the hypothesis that T334 phosphorylation promotes CtIP function in response to IR-induced damage. To evaluate the effect of pT334 on ssDNA resection activity, we next examined the formation of phospho-RPA32 foci in 53BP1-modified U2OS cells following irradiation by costaining pT334 with RPA32 (Figure 5D). We observed that RPA32 increased slowly at 8 hours and was significantly enhanced in pT334+ve cells compared with pT334-ve cells (Figure 5, E and F, and Supplemental Figure 4B). Together, these data suggest that phosphorylation of T334 facilitates ssDNA resection. BRCA1 promotes phosphorylation of 53BP1 and facilitates removal of 53BP1 from DSB sites to allow for resection and the transition from NHEJ to HR (28). Similar to CtIP and RPA32, costaining pT334 and BRCA1 showed an increase in BRCA1 with time following IR with a significant increase in pT334+ve cells compared with pT334-ve cells (Figure 6, A–C, and Supplemental Figure 5A). RAD51 complexes with additional proteins at various stages of HR (29), binds to ssDNA, and is essential for single strand invasion and subsequent HR. We next examined RAD51 nuclear foci formation in relation to T334 phosphorylation status using costaining with RAD51 antibody (Figure 6D). We also found a significant increase in late RAD51 foci in pT334+ve cells compared with pT334-ve cells at 8 hours following IR (Figure 6, E and F, and Supplemental Figure 5B). These experiments were repeated in MCF7 cells with similar results (Supplemental Figure 6, A and B, and Supplemental Figure 7, A and B). The observed foci kinetics of CtIP, RPA32, BRCA1, and RAD51 in response to DNA damage support the model that T334 phosphorylation is important for HR activity.

To confirm the role of T334 phosphorylation in HR, we employed the chromosomal integrated HR-specific reporter to investigate whether phosphorylation of T334 also confers an increase in HR repair. The stably integrated HR-GFP chromosomal reporter system contains GFP genes with varied mutations that require repair by HR for functional GFP expression (30). We analyzed enzymatic DSB-induced HR in *53BP1^–/–^* U2OS cells stably reconstituted with WT or T334A mutant (Figure 6G). Additionally, *BRCA1* was concurrently knocked down using *BRCA1*-specific siRNA (Supplemental Figure 7C). Consistent with the literature (31–33) and as shown in Figure 6G, *BRCA1* knockdown decreases HR repair efficiency compared with control, and loss of *53BP1* did not affect HR in BRCA1-intact cells but partially rescued HR efficiency in *BRCA1^–/–^* cells. Compared with cells expressing *53BP1*^+/+^, expressing T334A in *BRCA1* intact cells significantly decreased HR activity to the level similar to that in *BRCA1^–/–^* cells and further diminished HR in *BRCA1^–/–^* cells.

Together, these data support that T334 phosphorylation is critical for HR activity and phosphor-deficient T334A mutation impairs HR in both *BRCA1*-proficient and deficient cells. This is in contrast with the loss of *53BP1*, which requires *BRCA1* deficiency to rescue HR through alternative DNA end resection.

### T334 phosphorylation modulates 53BP1 interaction with PTIP and RIF1 and influences DSB repair dynamics.

To further investigate the findings in Figures 4 and 5 and uncover the mechanisms underlying the noncanonical phenotype, we examined the interaction between 53BP1 and the NHEJ mediators RIF1 and PTIP. These factors are essential for NHEJ regulation, as they are recruited to DNA damage sites and function to block end resection and restrict access of HR proteins. The recruitment of downstream NHEJ effectors by 53BP1 requires its physical interaction with both RIF1 and PTIP, thereby promoting NHEJ and suppressing HR-mediated repair (12, 34).

**Figure 7:**
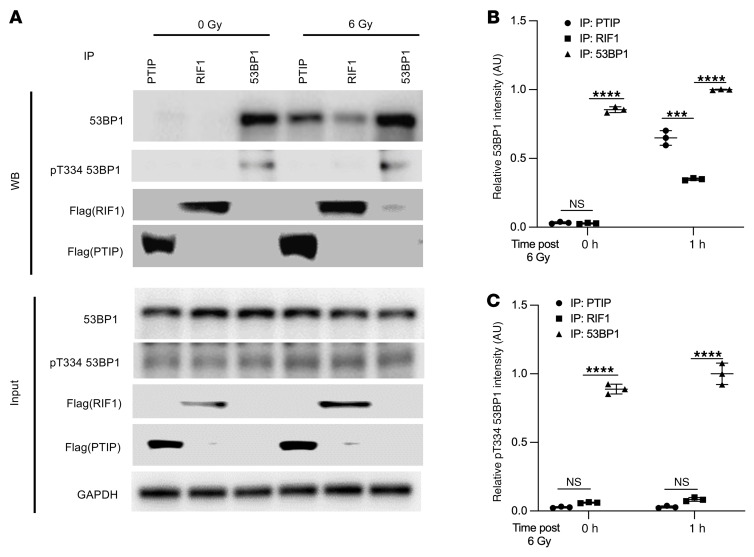
T334 phosphorylation modulates 53BP1 interaction with PTIP and RIF1 and influences DSB repair dynamics. (**A**) Coimmunoprecipitation assay of 53BP1 with PTIP and RIF1 in WT U2OS cells before and 1 hour after ionizing radiation (IR; 6 Gy). Relative intensities of (**B**) 53BP1 and (**C**) T334 phospho-53BP1 (pT334-53BP1) bands were quantified by ImageJ at 0 hours and 1 hour after IR (*n* = 3). Data represent mean ± SEM. Band intensities for 53BP1 and pT334-53BP1 were first normalized to their respective input levels and then further normalized to the input levels of PTIP and RIF1-FLAG signals across the 0 Gy and 6 Gy conditions. Values are mean ± SEM. Statistical significance was determined by 2-way ANOVA followed by Tukey’s multiple comparisons test. ***P < 0.001; ****P < 0.0001.

To determine whether T334 phosphorylation influences this interaction, we performed co-IP assays in WT U2OS cells before and 1 hour after IR. Following DNA damage, total 53BP1 showed increased binding to both PTIP and RIF1 (Figure 7, A and B). Notably, using a phospho-specific T334 antibody, we found that phosphorylated 53BP1 at T334 exhibited markedly reduced binding to PTIP and RIF1 compared with total 53BP1 (Figure 7, A and C), suggesting that T334 phosphorylation facilitates their dissociation.

**Figure 8:**
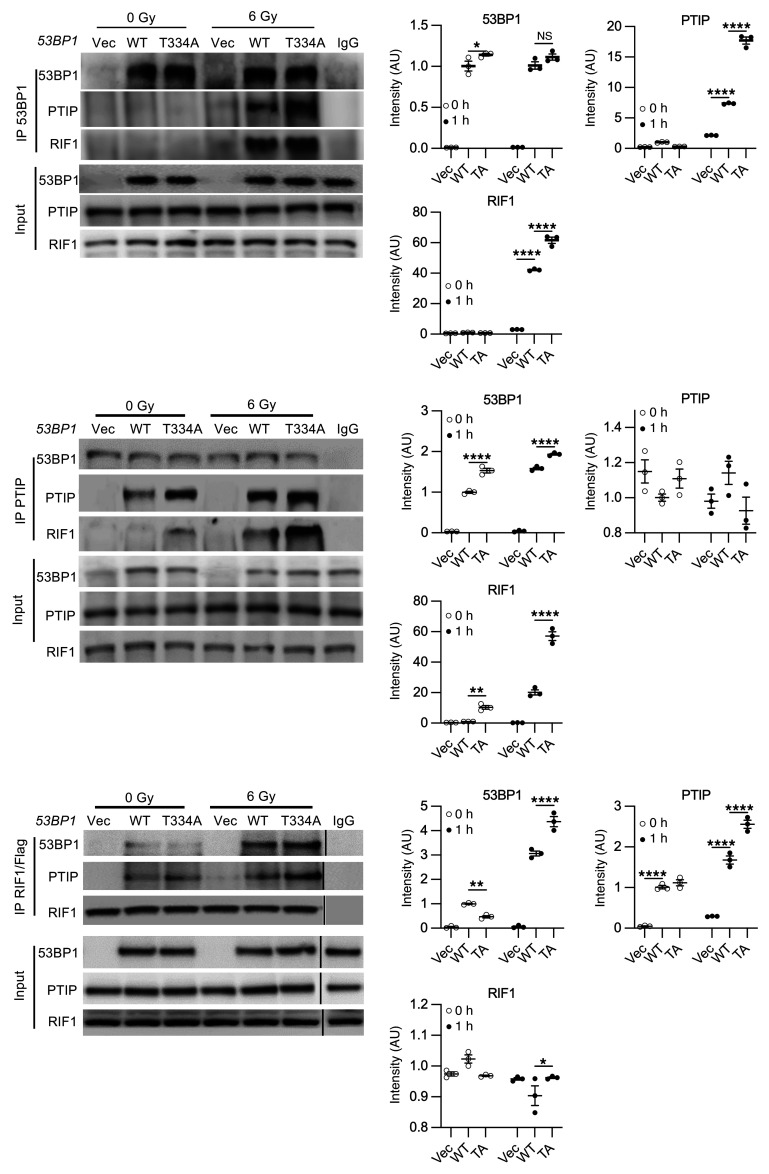
T334 phosphorylation modulates 53BP1 interaction with PTIP and RIF1 and influences DSB repair dynamics cont. Reciprocal coimmunoprecipitation assay of 53BP1 and PTIP and RIF1 with or without IR in WT and T334A reconstituted *53BP1^–/–^* U2OS cells before and after IR (6 Gy) (left panel). Quantification of Co-IP bands intensity at 0 hours and 1 hour. *n* = 3. Values are mean ± SEM. Statistical significance was determined by 2-way ANOVA followed by Tukey’s multiple comparisons test. *P < 0.05; ****P < 0.0001.

We then extended this analysis by performing reciprocal co-IP in *53BP1^–/–^* U2OS cells reconstituted with either WT or T334A mutant *53BP1* before and 1 hour after IR. As expected, IR enhanced the interaction of 53BP1 with PTIP and RIF1. However, the T334A mutant showed significantly greater binding to both PTIP and RIF1 compared with WT 53BP1 (Figure 8). These results indicate that phosphorylation at T334 is critical for releasing PTIP and RIF1 from 53BP1 following DNA damage, thereby relieving the block on DNA end resection and facilitating homologous recombination repair.

**Figure 9:**
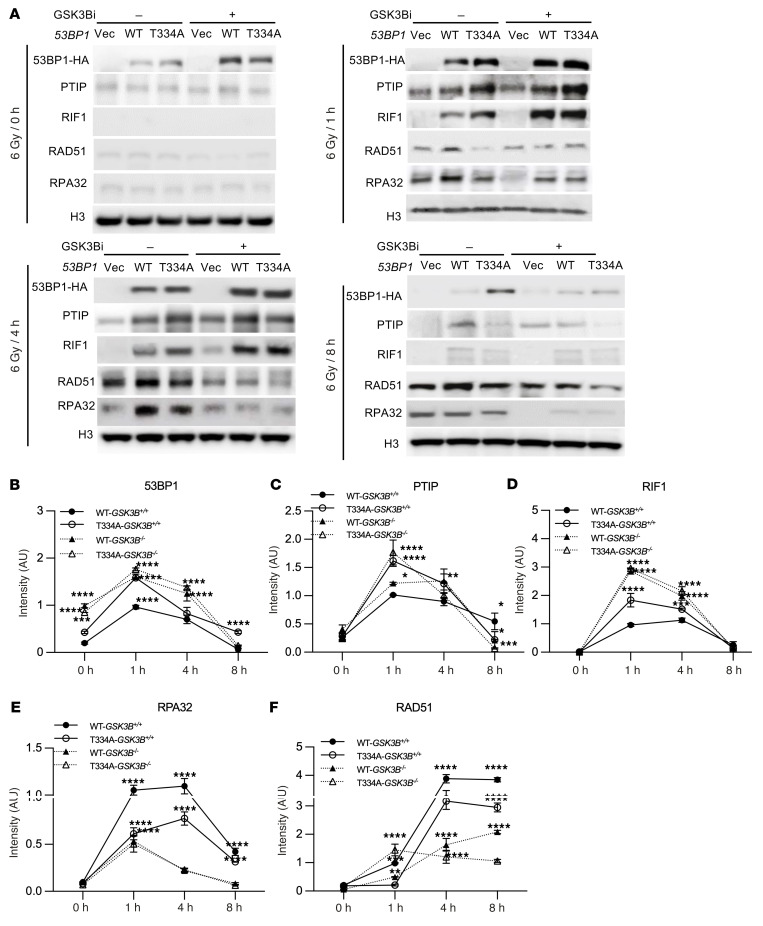
T334 phosphorylation modulates 53BP1 interaction with PTIP and RIF1 and influences DSB repair dynamics cont. (**A**) Chromatin binding assay in *53BP1^–/–^* U2OS cells reconstituted with HA-tagged WT or T334A 53BP1 or WT treated with GSK3B inhibitor and irradiated with 6 Gy to induce DNA damage. Quantification of chromatin binding with (**B**) 53BP1(HA), (**C**) PTIP, (**D**) RIF1, (**E**) RPA32, and (**F**) RAD51 at 0, 1, 4, and 8 hours following IR. *n* = 3. Values are mean ± SEM. Statistical significance was determined by 2-way ANOVA followed by Tukey’s multiple comparisons test. *P < 0.05; **P < 0.01; ***P < 0.001; ****P < 0.0001.

We next investigated the role of T334 phosphorylation in regulating the chromatin-binding kinetics of 53BP1, NHEJ factors PTIP and RIF1, and HR proteins RPA32 and RAD51. To this end, we performed chromatin fractionation followed by western blot analysis in *53BP1^–/–^* U2OS cells reconstituted with either WT or T334A mutant *53BP1*, with or without GSK3B inhibition following IR-induced DNA damage. Compared with WT 53BP1, disruption of T334 phosphorylation—via the T334A mutation and/or GSK3B inhibitor treatment—resulted in markedly enhanced chromatin retention of 53BP1 at all examined time points (Figure 9, A and B).

Consistent with this, PTIP and RIF1, 2 key NHEJ-associated factors known to inhibit homologous recombination by blocking DNA end resection, also showed significantly increased chromatin association in the absence of T334 phosphorylation, particularly between 1 and 4 hours after IR (Figure 9, A, C, and D). In contrast, recruitment of the HR proteins RPA32 and RAD51 was notably delayed and suppressed for up to 8 hours following DNA damage under conditions where T334 phosphorylation was disrupted (Figure 9, A, E, and F).

To validate these findings, we repeated chromatin fractionation and Western blot analysis in *53BP1^–/–^* MEF cells reconstituted with either WT or T334A mutant *53BP1*, with or without *Gsk3b^–/–^*, following IR-induced DNA damage. Similar results were observed (Supplemental Figure 8). Specifically, loss of T334 phosphorylation — either by the T334A mutation or *Gsk3b* knockout — led to increased chromatin binding of 53BP1, PTIP, and RIF1 between 1 and 4 hours after IR (Supplemental Figure 8, A–D), while RPA32 and RAD51 recruitment remained significantly impaired up to 8 hours after damage (Supplemental Figure 8, A, E, and F).

These findings suggest that phosphorylation at T334 plays a regulatory role in coordinating the timely dissociation of PTIP and RIF1 from DSBs, thereby directly or indirectly facilitating access of RPA32 and RAD51 and promoting HR repair.

### GSK3B-53BP1 axis controls HR-mediated DSB repair and determines synthetic lethality response of tumor cells to PARP1 inhibition independent of BRCA1 status.

**Figure 10:**
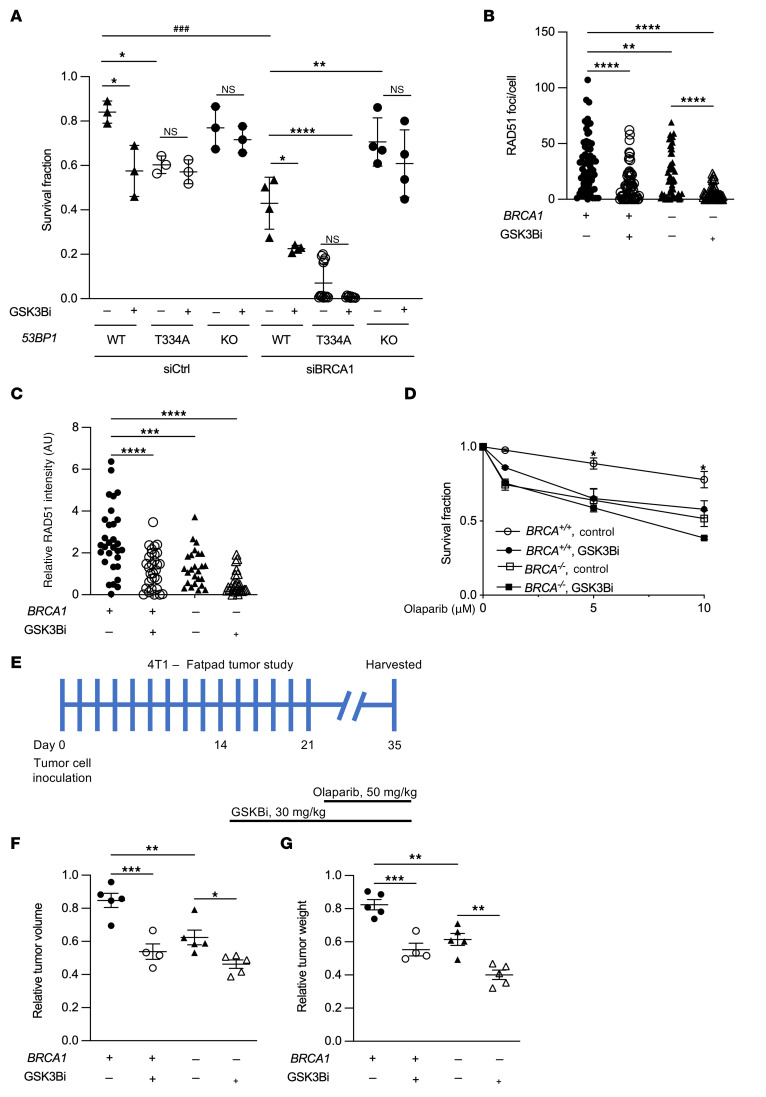
T334 phosphorylation of 53BP1 is critical for chromosomal HR efficiency and determines synthetic lethality of PARPi independent BRCA1 status. (**A**) *53BP1^–/–^* U2OS cells reconstituted with WT or T334A 53BP1 with or without BRCA1 knockdown and in the presence or absence of GSK3B inhibitor following PARPi were assessed by colony formation assay with survival fractions calculated. *n* = 3. (**B**) Number of RAD51 foci per cell and (**C**) relative intensity of RAD51 foci in *BRCA1*-deficient MDA-MB-436 cells with or without BRCA1 reconstituted and with or without GSK3B inhibitor incubated with 15 μM of olaparib. *n* = 3. (**D**) *BRCA1*-deficient MDA-MB-436 cells with or without BRCA1 reconstituted and with or without GSK3B inhibitor were incubated with the indicated concentrations of olaparib, and survival fractions were assessed with colony formation assay. *n* = 3. (**E**) Mouse 4T1 in vivo tumor study scheme. (**F**) Tumor volumes and (**G**) weights in C57BL6 mice injected with 4T1 cells with or without *BRCA1* knock down in the fat pad. Mice were treated with Lithium (30 mg/kg) and/or Olaparib (50 mg/kg). Values are mean ± SEM. Statistical significance was assessed using 1-way ANOVA followed by Tukey’s multiple comparison test and 2-way ANOVA with Bonferroni’s correction. ^###^*P* < 0.001; **P < 0.01; ***P < 0.001; ****P < 0.0001. * indicates comparing same biological group under treatment. ^#^ indicates comparing 2 different biological groups under treatment.

HR deficiency, such as in BRCA1/2-mutated cancer cells confers synthetic lethality to PARPi. Loss of *53BP1* has been implicated in the development of PARPi resistance by rescuing HR-deficient cells and activating alternative DNA repair pathways (35). Olaparib is a PARPi showing encouraging success in the treatment of *BRCA1*-deficient cancers in clinic. Given the described role of GSK3B-mediated phosphorylation of T334 in facilitating HR repair regardless of *BRCA1* status (Figure 6G), we asked whether interruption of T334 phosphorylation would sensitize cancer cells to the PARPi Olaparib. In an in vitro cell culture setting, cell sensitivity to PARPi-induced synthetic lethality was assessed using colony formation assay. *BRCA1^–/–^* significantly decreased survival compared with *BRCA1*-proficient U2OS cells, as expected. In *BRCA1*-proficient U2OS cells, we observed that disruption of T334 phosphorylation genetically with T334A mutation or pharmacologically with GSK3Bi (SB216763) resulted in significantly decreased survival in response to Olaparib compared with control cells (Figure 10A). Importantly, pharmacologic GSK3B inhibition or expression of T334A further significantly sensitized *BRCA1*-deficient cells to Olaparib. GSK3Bi did not further affect Olaparib cytotoxicity in T334A cells, indicating that T334 is the required site phosphorylated by GSK3B for 53BP1 to facilitate HR repair. In addition, GSK3Bi-mediated suppression of HR and sensitization to PARPi were not observed in *53BP1^–/–^* cells (Figure 10A), suggesting that reduction of HR by GSK3B inhibition is dependent on its phosphorylation of 53BP1 and further supports the GSK3B-53BP1 signaling axis in DSB repair.

To confirm that the function of the GSK3B-53BP1 signaling pathway in HR is not an isolated phenotype in U2OS cells, we examined HR activity and sensitivity to PARPi in more cancer cell lines. We used an isogenic pair of human breast cancer cell lines MDA-MB-436, which are inherently *BRCA1* deficient, reconstituted with or without wt*BRCA1*. We showed that pharmacological GSK3B inhibition resulted in a significant decrease of HR activity assessed by RAD51 foci formation (Figure 10, B and C, and Supplemental Figure 9A) and increased sensitivity to Olaparib-induced cytotoxicity compared with control in both *BRCA1*-proficient and deficient MDA-MB-436 cells (Figure 10D). We observed a similar effect by pharmacological GSK3B inhibition using SB216763 on PARPi sensitization in murine breast cancer cell 4T1 regardless of *BRCA1* status (Supplemental Figure 9B) and *BRCA1*-proficient human breast cancer cells MCF7 (Supplemental Figure 9C). In contrast, GSK3B inhibition did not significantly affect sensitivity of cancer cells to IR-induced killing in MCF7 and U2OS cells (data not shown).

We next examined whether in vivo pharmacological inhibition of GSK3B using lithium affects PARPi-induced tumor control in mouse allogenic orthotopic breast tumor. Mouse 4T1 cells with and without siRNA-mediated *BRCA1^–/–^* (Supplemental Figure 9B top panel) were inoculated in the fat pad of mice, Olaparib was administrated alone or in combination with GSK3Bi lithium, and the tumor volumes and weights were measured at day 14 after Olaparib (Figure 10E and Supplemental Figure 9D). As shown in Figure 10, F and G, *BRCA1^–/–^* tumor showed a decrease in tumor volume and weight in response to Olaparib, as expected. Inhibition of GSK3B sensitized tumor response to Olaparib with a significant decrease in tumor volume and weight compared with tumors treated with Olaparib alone in both *BRCA1*-proficient as well as *BRCA1*-deficient 4T1 tumors.

### Targeting GSK3B-53BP1 axis to enhance PARPi-induced synthetic lethality of cancer cells depends on functional 53BP1.

**Figure 11:**
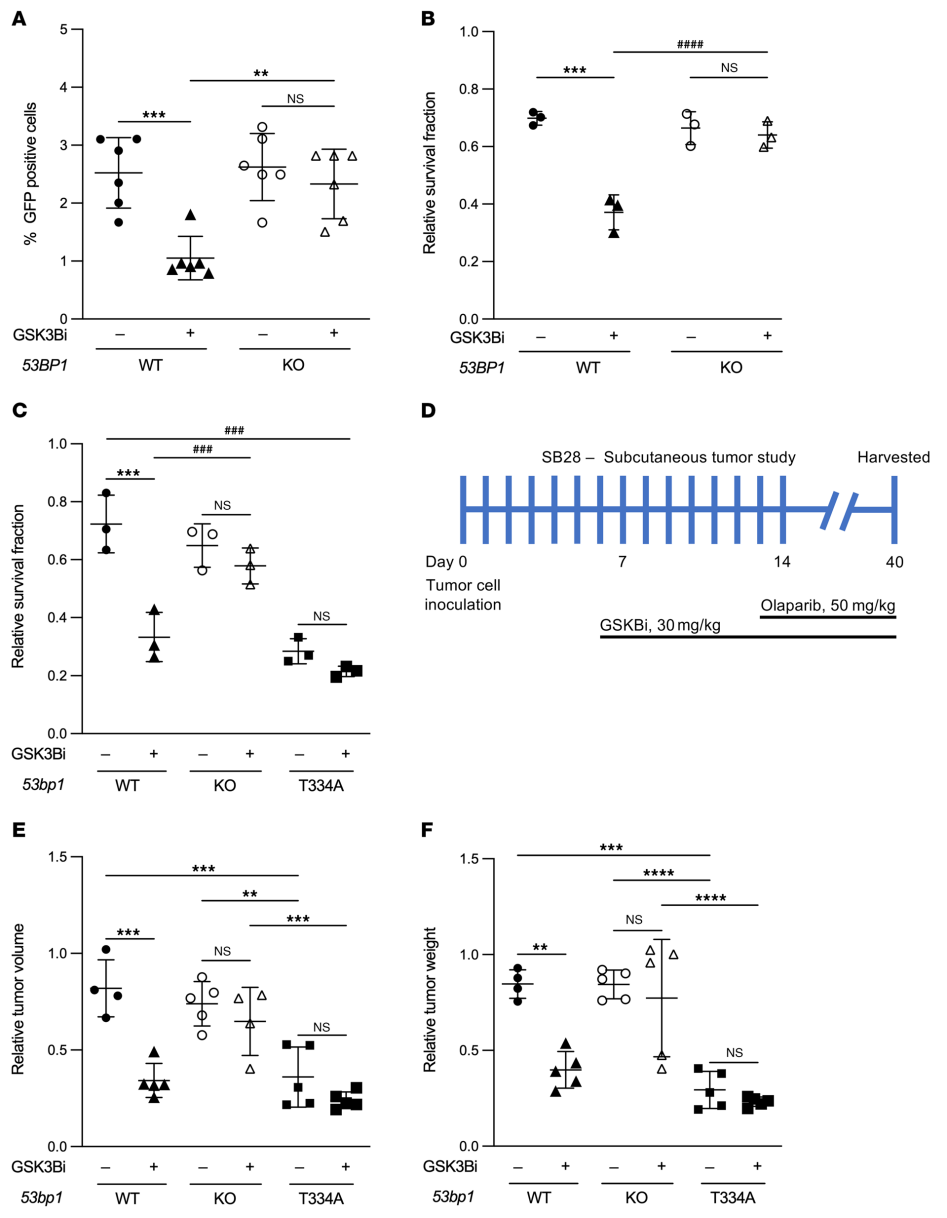
GSK3B inhibition-mediated sensitization to PARPi requires functional 53BP1. (**A**) Assessment of HR of DSB quantified by % GFP positivity measured through flow cytometry in *53BP1^–/–^* MCF-7 cells with reconstitution of WT 53BP1 in the presence or absence of GSK3B inhibitor. DSBs were induced by adenoviral delivery of I-SceI, and the ability of cells to resolve DSBs by HR was assessed 48 hours later. *n* = 3. (**B**) Relative survival fractions of MCF-7 cells in the presence or absence of GSK3B inhibitor in response to the PARPi, Olaparib. *n* = 3. (**C**) Relative fractions of SB28 cells in the presence or absence of GSK3B inhibitor subjected to the PARPi Olaparib. *n* = 3. (**D**) SB28 subcutaneous tumor study scheme. (**E**) SB28 subcutaneous tumor volumes and (**F**) weights in C57BL6 mice subcutaneously injected with *53BP1^–/–^*, *53BP1^+/+^*, or *53BP1* T334A SB28 cells in the flank region then treated with Lithium (30 mg/kg) and/or Olaparib (50 mg/kg). Values are mean ± SEM. Statistical significance was assessed using 2-way ANOVA with Bonferroni’s correction. ^###^*P* *<* 0.001; ^####^*P* *<* 0.0001; *P *<* 0.05; **P < 0.01; ***P *<* 0.001; ****P *<* 0.0001. * indicates comparing same biological group under treatment. ^#^ indicates comparing 2 different biological groups under treatment.

53BP1 has been implicated in the development of PARPi resistance by rescuing HR-deficient cells and activating alternative DNA repair pathways (35). Interestingly, the T334A mutant exhibits increased recruitment and retention at DSB sites (Figure 2, A and B) compared with WT 53BP1. We propose that, in contrast with loss of 53BP1 where DSB ends are accessible for resection, deficiency in T334 phosphorylation makes 53BP1 remain abnormally at DSB ends, which interferes with the dynamic binding of repair factors at the DSB sites and obstructs end resection and HR repair initiation. In support of this model, we have observed that expression of *53BP1^–/–^* rescued HR activity and rendered PARPi resistance in *BRCA1^–/–^* U2OS cells. This contrasts with severely impaired HR and decreased cell survival to PARPi, shown with expression of phosphor-deficient mutation of T334A (Figure 10A). Intriguingly, we found that GSK3Bi-induced suppression of HR activity and sensitization to PARPi cytotoxicity occurred only in breast cancer MCF7 cells with intact 53BP1, but no longer affected HR efficiency and PARPi-induced cytotoxicity in *53BP1^–/–^* MCF7 cells (Figure 11, A and B). To validate this further, we compared the effect of GSK3Bi on sensitization of SB28 mouse glioma tumor cells with *53BP1*-null versus T334A to PARPi-induced cytotoxicity in in vitro cell survival and in in vivo C57BL6 mouse subcutaneous tumor control. We again observed that GSK3B inhibition significantly reduced the *wt53BP1* SB28 cell survival fraction (Figure 11C) and inhibited tumor growth, measured by tumor volume and weight at day 26 following PARPi (Figure 11, D–F, and Supplemental Figure 10). In contrast, GSK3B inhibition had no effect on Olaparib-induced cytotoxicity in vitro and tumor control in vivo when SB28 cells lost *53BP1^–/–^*. Intriguingly, the expression of T334A *53BP1* in SB28 dramatically enhanced response to Olaparib-induced cell killing and tumor control without further effect on PARPi sensitivity with GSK3B inhibition (Figure 11, C–F, and Supplemental Figure 10). Together, these data indicate the different roles of *53BP1*-null versus the 53BP1 T334A mutation on regulation of DSB repair; furthermore, overcoming PARPi resistance through pharmacological targeting of the GSK3B-53BP1 axis requires functional 53BP1 in tumor cells.

## Discussion

In this study, we identify a novel GSK3B-53BP1 axis and illustrate that GSK3B phosphorylates 53BP1 at T334. 53BP1 phosphorylation at T334 controls its function in canonical NHEJ through regulation of 53BP1 recruitment to and retention at DNA DSB sites as well as controls the dynamics at DSB lesions of downstream mediators, RIF1 and PTIP. Unexpectedly, T334 phosphorylation disassociates its binding with PTIP/RIF1, removing them from damaged chromatin, either directly or indirectly enhancing ssDNA resection, and promoting HR repair of DSBs. Importantly, genetic and pharmacological destruction of GSK3B-mediated T334 phosphorylation leads to HR repair deficiency and makes cancer cells susceptible to synthetic lethality of PARPi therapy independent of *BRCA1* status. Lastly, overcoming PARPi resistance through pharmacological targeting the GSK3B-53BP1 axis requires functional 53BP1 in tumor cells.

53BP1 plays key roles in DNA damage response and repair, and the regulatory mechanisms of its function, including recruitment, localization (36, 37), and chromatin binding (38–40), have been well documented. A variety of posttranslational modifications have been described in the regulation of 53BP1, including acetylation, which inhibits NHEJ (41), phosphorylation to prevent localization to chromatin (42), and stabilization of 53BP1 through methylation (43). Despite lacking enzymatic activity, 53BP1 has several well-characterized interaction domains that play pivotal roles in its function (44). Interestingly, T334 is not located in one of these sites. In addition to the 28 S/T-Q sites, tandem Tudor domains and BRCT repeats are important structural elements in 53BP1. Tudor domains bind mono- and demethylated H4K20 as an essential step in 53BP1 recruitment to DSB sites (40), and BRCT domains have been shown to interact with multiple factors, including p53 (45), chromatin modulator EXPAND1 (46), and the RAD50 component of MRN complex, which stimulates ATM activity (47). Whether additional sites play a role in the GSK3B-53BP1 signaling axis warrants further investigation.

This study identifies a unique inhibitory effect of GSK3B-mediated phosphorylation of 53BP1 and counters the general paradigm that 53BP1 is activated through phosphorylation and inactivated by dephosphorylation. Extensive investigation has shown phosphorylation to be integral to activation of 53BP1 with implications on both class-switch recombination and DNA repair (9, 17, 48–50). For example, the 28 terminal S/T-Q sites are phosphorylated by ATM to promote the binding of RIF1 and PTIP. Moreover, ATM-mediated phosphorylation of 53BP1 is required for RIF1 recruitment to damaged chromatin (12, 51–54). Conversely, dephosphorylation is known to inhibit 53BP1, as illustrated by the BRCA1-mediated promotion of HR through dephosphorylation of 53BP1 (55). The selective inhibitory effect on NHEJ with concurrent promotion of HR identified in our study is what we believe to be a novel role of GSK3B in contrast with the well-documented inhibitory effects of GSK3B-dependent phosphorylation on multiple proteins, including the first identified substrate, glycogen synthase. The underlying molecular mechanism for this effect on HR is well worth investigating in future studies.

As recent studies have reported, 53BP1 plays a role in DSB repair choice and its anti-HR function is precisely controlled. Our data supports 53BP1’s canonical anti-HR function and agrees with the working model in the literature that fine tuning and coordination of HR is needed to repair the DSB lesion. Several regulators and signaling pathways have been reported to modify 53BP1’s anti-HR function through different mechanisms (13, 48, 56, 57). Based on the impact of T334 on 53BP1 interactions with PTIP and RIF1 (Figures 8 and 9), its kinetics at DNA damage sites (Figures 2 and 4–6), and its effects on CtIP, RPA32, BRCA1, RAD51 foci, and HR repair (Figures 5 and 6), we propose a working model: unlike ATM-mediated phosphorylation, which promotes 53BP1 binding, retention, and end protection, GSK3B-mediated phosphorylation at T334 fine-tunes 53BP1 dynamics by limiting its retention at DSBs, facilitating the release of PTIP and RIF1, and enabling timely initiation of homologous recombination. This mechanism parallels the regulation of 53BP1 by protein phosphatase 5–mediated dephosphorylation (57) and its suppression of 53BP1’s anti-HR functions. New questions are also raised from this study, such as, what is the function of T334 phosphorylated 53BP1 remaining at DSB site at 8 hours after HR initiation? Recent studies suggest that posttranslationally modified 53BP1 at DSBs during HR is not inert but plays regulatory, architectural, and checkpoint roles that fine-tune repair fidelity and coordinate repair (9, 58). Unanswered questions remain for future studies including whether T334-phosphorylated 53BP1 scaffolds ATM-dependent DDR signaling, sustains the G2/M checkpoint, and promotes chromatin reorganization. These findings identify a dynamic role of 53BP1 as a phosphorylation-regulated coordinator of DSB repair.

Our study identifies the physical and functional interaction of GSK3B and 53BP1 and illustrates the role of GSK3B-mediated phosphorylation of 53BP1 at T334. Although we identify a single site, GSK3B may interact with 53BP1 at additional sites. GSK3B-mediated phosphorylation of 53BP1 has also been shown at S166, although with marked differences in effect. In contrast with our findings, phosphorylation promoted DSB repair, and inhibition of GSK3B induced glioblastoma cell apoptosis. No distinction between NHEJ- and HR-mediated DSB repair was made (20).

BRCA1 is a well-known tumor suppressor targeted by systemic therapy in multiple cancer types and is often mutated in familial breast and ovarian cancers. Mechanistically, BRCA1 contributes to HR through resection of DSBs to generate ssDNA and loading of RAD51 onto ssDNA (59). Conversely, 53BP1 suppresses HR by blocking DSB end resection (29, 60), and loss of 53BP1 rescues HR in *BRCA1*-deficient cells. Intriguingly, our data revealed that the loss of GSK3B-dependent T334 phosphorylation, different from loss of 53BP1, significantly decreases HR in *BRCA1*-deficient and -proficient cells. The precise mechanism through which this occurs and the role of T334 on DSB resection will be the subject of future studies.

Here, we show that phosphorylation of 53BP1 by GSK3B promotes HR and renders PARPi toxicity independent of BRCA1 status. Clinically, PARP inhibitors exploit the HR defect in *BRCA1*-deficient cancers and are a highly effective systemic treatment with limited toxicity. However, *BRCA1*-mutated tumors can develop resistance and subsequent progression of disease. Multiple mechanisms of resistance have been identified, including genetic reversion of the *BRCA1* mutation (61), loss of 53BP1 (32, 62, 63), and protection of the DNA replication fork (35, 64). PARPi resistance due to 53BP1 loss appears to result from partial restoration of impaired HR in *BRCA1*-deficient cells (32). Additionally, *BRCA1*-deficient mouse mammary tumors that became resistant to Olaparib after initial response were found to have lost 53BP1 and partially recovered HR (33). Clinical application of these findings is limited by the fact that we cannot selectively manipulate *53BP1* genetically, but pharmacologic manipulation of 53BP1 function, as seen in our data, could have implications on tumor sensitivity to radiation and PARPi. More importantly, while *BRCA1/2* deficiency is rare, T334 represents a potential target to induce HR deficiency in tumor cells and therefore expand PARPi indication to more patients. Moreover, the GSK3B inhibitor currently under phase 1/2 study (NCT03678883) represents a potential pharmacologic agent that could be quickly integrated into clinical practice.

In summary, our results identify what we believe to be a novel signaling pathway involving 53BP1 and GSK3B, whereby GSK3B-dependent phosphorylation inhibits 53BP1 at T334. These preclinical data provide a potential target for enhancing DNA damage-based cancer treatments.

## Methods

### Sex as a biological variable.

Sex was not considered as a biological variable in the subcutaneous SB28 tumor model, but only female mice were examined for Orthotropic 4T1 tumor engraftment because the disease modeled is only relevant in females.

### Plasmids and siRNAs, CRISPR/Cas9-mediated knockout.

HA-53BP1 and deletion mutant expression vectors were previously described (42). The Flag-53BP1 WT and mutants were verified by Sanger sequencing and were subcloned into Gateway compatible destination vectors using Gateway cloning technology (Invitrogen). Mutations for 53BP1, T334A, and T334D were created using QuikChange site-directed mutagenesis kit (Agilent Genomics) according to the manufacturer’s instructions. Mission siRNA targeting GSK3B were purchased from Sigma. Clone targeting specific GSK3B sequence (5′-CATGAAAGTTAGCAGAGATAA-3′) was used for knock down, and a nonspecific targeting sequence (5′-CGAGAAGAAAGATGAGGTCTA-3′) was used as a control. For lentiviral infection, lentiviral expression vectors pMD2.G and psPAX2 were cotransfected into HEK293T cells in a 4:3:1 ratio using Fugene according to the manufacturer’s instructions. Viral particles were collected 48 hours after transfection and applied to target cell lines in the presence of 8 μg/ml polybrene (Sigma). For BRCA1 knock down, Signal Silence BRCA1 siRNA was purchased from Cell signaling, # 12519. 53BP1 knockout cells used in this study were generated using the CRISPR/Cas9 method (58). Two specific gRNAs were designed (gRNA1 – ATAATTTATCATCCACGTCT and gRNA2 – CATAATTTATCATCCACGTC) and subcloned into pSpCas9 (BB)-2A-Puro (Addgene IDs: 48139). Gene knockout was performed as previously described (42). In brief, cells were transfected with the mammalian expression vectors containing gRNAs using Fugene reagent according to the manufacturer’s instructions. Transfected cells were selected for with puromycin for 7 days and then seeded into 96-well plates to obtain single colonies. The cells derived from single colonies were then screened by Western blotting, and immunofluorescence and sequences were verified by sequencing.

### Cell culture.

HEK293T, U2OS, MCF7, MDA-MB-436, and HN33 cells, which were derived from the fusion of primary hippocampal neurons of postnatal day 21 mice and the N18TG2 neuroblastoma cell line, were purchased from American Type Culture Collection. SB28 cells were obtained from Hideho Okada, UCSF, San Francisco, California, USA. All were cultured in Dulbecco’s modified Eagle’s medium (DMEM) and supplemented with 10% FBS, 100 U/ml penicillin, and 100 μg/ml streptomycin at 37°C with 5% CO_2_. U2OS 53BP1 CRISPR-KO and U2OS-KO cell lines rescued with T334A or T334D mutants of 53BP1 were generated. GSK3B WT and KO MEFs were obtained from J. Woodgett, the Lunenfeld-Tanenbaum Research Institute, and Mount Sinai Hospital, Toronto, Ontario, Canada. 4T1 WT and Brca1-deficient mouse mammary tumor cells were obtained from Martin Cannon, the University of Arkansas for Medical Sciences (UAMS) and cultured in Roswell Park Memorial Institute Medium (RPMI) and supplemented with 10% FBS, 100 U/ml penicillin, and 100 μg/ml streptomycin at 37°C with 5% CO_2_. Transfection was carried out using Fugene HD (Promega) and Polyethylenimine (PEI) (Sigma Aldrich) according to the manufacturer’s instructions.

### Immunoblotting, immunostaining, and antibodies.

Cells were washed twice with cold PBS, lysed using lysis buffer (150 mM NaCl, 50 mM Tris, pH 8.0, 5 mM EDTA, 1.0% Nonidet P-40, 0.1% SDS, 0.5% sodium deoxycholate), protease inhibitors (Sigma-Aldrich, catalog P8340), and phosphatase inhibitor cocktail (Roche, catalog 4906837001), and analyzed by SDS-PAGE. Primary antibodies used in the current study were Flag M2 (Sigma, F1804), HA (Abcam, AB18181), 53BP1 (Novus Biologicals, NB100-304), γH2AX (Ser139), clone JBW301 (Millipore-Sigma, 05-636), PTIP (Abcam; ab70434), actin (Santa Cruz Biotechnology, SC-47778), GAPDH (Proteintech, 6004-1), Histone H3 (Abcam, ab1791), RIF1 (Bethyl Laboratories, A300–568A), P threonine (Cell signaling, 9386S) P GSK3 (Tyr216) (Santa Cruz Biotechnology, sc-135653), B-Catenin (6B3; Cell signaling, 9582), BRCA1 (Santa Cruz Biotechnology, SC-6954), Anti-RPA32/RPA2 (phospho S33) (Novus Biologicals, NB100–544), CtIP (Santa Cruz; sc-271339), and Rad51 (Abcam; ab133534). Secondary antibodies for Western blotting, HRP-linked anti-rabbit IgG and HRP-linked anti-mouse IgG were purchased from Cell Signaling (0704 and 0706). For immunofluorescence, Alexa Fluor 594 goat anti-mouse (Invitrogen # A-11012) and Alexa Fluor 488 goat anti-rabbit (Invitrogen # A-11008) and were used. The custom pT334 antibody was generated by Bethyl Laboratories against phosphorylated T334 peptide.

### Coimmunoprecipitation and Western blotting.

Cells were transfected with SFB-fused proteins as indicated and harvested with NETN buffer with nuclease at 4°C for 30 minutes. The lysates were incubated with protein A/G magnetic beads (Cell Signaling, 70023, 70024), precoated with 2 μg of IgG1 or indicated antibodies, washed with NETN buffer with nuclease at 4°C for 30 minutes, and incubated with 0.5 ml of cell lysates overnight at 4°C. After washing 3 times in lysis buffer, the immunoprecipitated complexes were eluted with 1× Laemmli buffer and resolved by SDS-PAGE. They were transferred to PVDF membranes, immunoblotted with antibodies as indicated, and imaged using BioRad ChemiDoc MP.

### Chromatin bound protein assay.

Chromatin fractions were prepared as described previously (59, 60). Briefly, cells were collected and washed once with PBS. Cell pellets were subsequently resuspended in NETN buffer. Lysates were removed by centrifugation at 24000 xg at 4°C for 10 minutes, and the pellet was resuspended in chromatin extraction buffer (NETN, with 100 mM MgCl and 5 μl turbo nuclease). The soluble fraction was collected for further analysis.

### Laser-induced microirradiation.

Cells were seeded on 35 mm glass bottom plates. Laser-induced microirradiation was carried out using a Nikon Ti2 inverted fluorescent microscope and C2+ confocal system. Cells were damaged using a fixed wavelength of 405-nm laser at 60% power. Live cell images were captured in 1-minute intervals following the damage. The fluorescence intensity of GFP-tagged protein in the damaged region was measured and normalized with an undamaged area from the same cell. Quantification analyses were done using the ImageJ software.

### Immunocytochemistry and Immunofluorescence microscopy.

For DNA repair experiments, cells were cultured and mounted onto sterile glass slides. Following different treatments, cells were exposed to either mock IR or 6 Gy IR using an XRAD-320 X-ray machine (Precision X-Ray) delivering 2.04 Gy/min at 80 kVp. IHC for 53BP1, γ-H2AX, Rad51, RIF1, and PTIP (flag), P-T334, RPA32, and Coimmunostaining experiments of P-T334 with HA, Rad51, RPA32, RAD51, CTIP, or BRCA1, were performed as previously described (58). Briefly, cells were seeded on poly-L-lysine coated coverslips (BD Biosciences) for 24 hours. Coverslips were washed in PBS and fixed in 3% paraformaldehyde for 15 minutes at room temperature, then permeabilized using 0.5% Triton X-100 solution. For foci detection, samples were preextracted with 0.5% Triton X-100 solution for 5 minutes prior to paraformaldehyde fixation. Samples were incubated with the indicated primary antibodies for 2 hours and washed and incubated with secondary antibodies and DAPI for 30 minutes at room temperature. Samples were then mounted onto glass slides with antifade solution [Sigma, P6001] in 90% glycerol in PBS. Samples were visualized and captured using a Ti-2 inverted C2+ confocal microscope. Recruitment intensity was analyzed by ImageJ software. Total cells were counted under a fluorescent microscope (×400 objective; Carl Zeiss), and cells containing greater than 10 foci were considered positive. At least 500 cells were counted.

### Colony formation assays.

Cells were seeded at a density of 500 or 1,000 cells per well in a 6-well plate in triplicates. Cells were treated as designated 24 hours after plating. After 10–14 days, cells were fixed and stained with Crystal Violet Staining Solution (0.5%) (Sigma-Aldrich), washed, and dried, the visible colonies were manual counted.

### Cytotoxicity assay.

Cells were plated in triplicates and incubated with vehicle or the desired compound (SB-216763 10 μM /or olaparib; 1 μM, 5 μM, 10 μM) for 48 hours. After that, cells were seeded at 4,000–8,000 cells /well in 96 well plates in cell culture medium containing 5% FBS. Following the addition of chemical compounds (SB-216763 10 μM /or olaparib; 1 μM, 5 μM,10 μM each), the plates were placed in the IncuCyte –SX1 Live Cell Analysis System (Sartorius Inc.) where the confluence and real time images were captured every 2 hours for 96 hours for the entire experiment. The confluence of each group of cells was evaluated and plotted with the IncuCyte-2022 software.

### HR and NHEJ DNA repair reporter assays.

The DR-GFP reporter system used to determine repair of I-SceI-induced DSBs via HR has been described previously (61).We transfected the DR-GFP plasmid into human U2OS cells (U2OS/DR-GFP) and established a stably transfected clone by selection with puromycin (5 μg/mL). Briefly, this reporter contains a cassette of 2 differently mutated GFP genes in direct repeat. One repeat contains a single I-SceI site, while the other copy is a truncated GFP fragment. I-SceI generates a DSB that, if repaired by HR, results in a complete GFP open reading frame. Translation of GFP was measured by flow cytometry (FACSort, Becton-Dickinson). Chromosomal DSBs were induced by infecting U2OS/DR-GFP cells adenovirus expressing I-SceI (pCBASceI) in the presence of 8 μg/ml of polybrene (62). Cells were allowed to recover for 48 hours after infection and were analyzed GFP expression by flow cytometry analysis using The BD Accuri C6 plus Flow Cytometer (BD Biosciences). 40,000 events were acquired per sample.

### Orthotropic 4T1 tumor engraftment.

Ten-week-old male BALB/c mice (22–25 g) were purchased from Jackson Laboratory. Before implantation, 4T1-WT reconstituted or BRCA1-deficient cells were grown for 2 days. Cells from the second passage were collected, counted, and resuspended in sterile PBS. Mice were anesthetized using isoflurane and 2 × 10^5^ cells per mouse 4T1 cells were suspended in 50 μL of PBS and injected into the mammary fat pad with a 30-gauge blunt-ended 0.5-inch needle. Mice were treated with LiCl 30 mg/kg or Olaparib 50 mg/kg or both. After 4T1 tumors become palpable, tumors were measured semiweekly using digital calipers to calculate their volume [(length × width^2^) / 2]. Tumors were harvested after 40 days and tumor weights were measured.

### SB28 cells subcutaneous flank injections.

7–8 week-old C57BL/6J female mice were acquired from Jackson Laboratory and acclimated for 7 days prior to injection date. 53BP1 WT or T334A SB28 cells were injected subcutaneously into the right flank region of the mice (day 0). Tumor-bearing mice were monitored for tumor growth 1–2 times weekly and tumor volume of subcutaneous flank tumor-bearing mice was measured via digital caliper measurement. Tumor volume was calculated by the modified ellipsoid formula (length × width^2^)/2.

### Statistics.

Data are represented as mean ± SEM and SD from at least 3 independent experiments. Analyses were performed using Graph Pad Prism by 2-tailed Student’s *t* test, 1- and 2-way ANOVA or χ^2^, where specified. Significance was reported at *P* < 0.05 (65).

### Study approval.

Mice were housed in the University of Arkansas for Medical Sciences Animal Research Facility. All experimental methods were conducted under a protocol (IPROTO202200000405) approved by the Institutional Animal Care and Use Committee of the University of University of Arkansas according to National Institutes of Health guidelines. Endpoint criteria by which mice were euthanized were defined per our approved IACUC protocol as exhibiting greater than 15% weight loss from baseline.

### Data availability.

Values underlying graphed data can be accessed in the Supporting Data Values file.

## Author contributions

HSA contributed to conduction of experiments and data analysis and interpretation. SAW contributed to data analysis, interpretation, and manuscript preparation. Order of co–first authorship was determined by contribution to experiments. HSA was listed first as she initiated the project. RS and MP contributed to conduction of experiments and data collection. FX led this study, contributed to hypothesis, experimental design, data analysis and interpretation, and manuscript preparation.

## Funding support

This work is the result of NIH funding, in whole or in part, and is subject to the NIH Public Access Policy. Through acceptance of this federal funding, the NIH has been given a right to make the work publicly available in PubMed Central.

- This study was supported by NIH grants R01 CA188500, R01 CA163838, and R01 CA247947, University of Arkansas for Medical Sciences science seeding grant, and vouchers to F.X and HSA.

## Supplementary Material

Supplemental data

Unedited blot and gel images

Supporting data values

## Version 1 11/17/2025

Electronic publication
