# Supplementary material for: GSK3B directs DNA repair choice and determines tumor response to PARP1 inhibition independent of BRCA1
Source: J Clin Invest. 2025 Nov 17;135(22):e189956. doi: 10.1172/JCI189956 (PMC12618078; doi:10.1172/JCI189956)
Supplement: Supplemental data [file jci-135-189956-s132.pdf]

Supplemental Figures

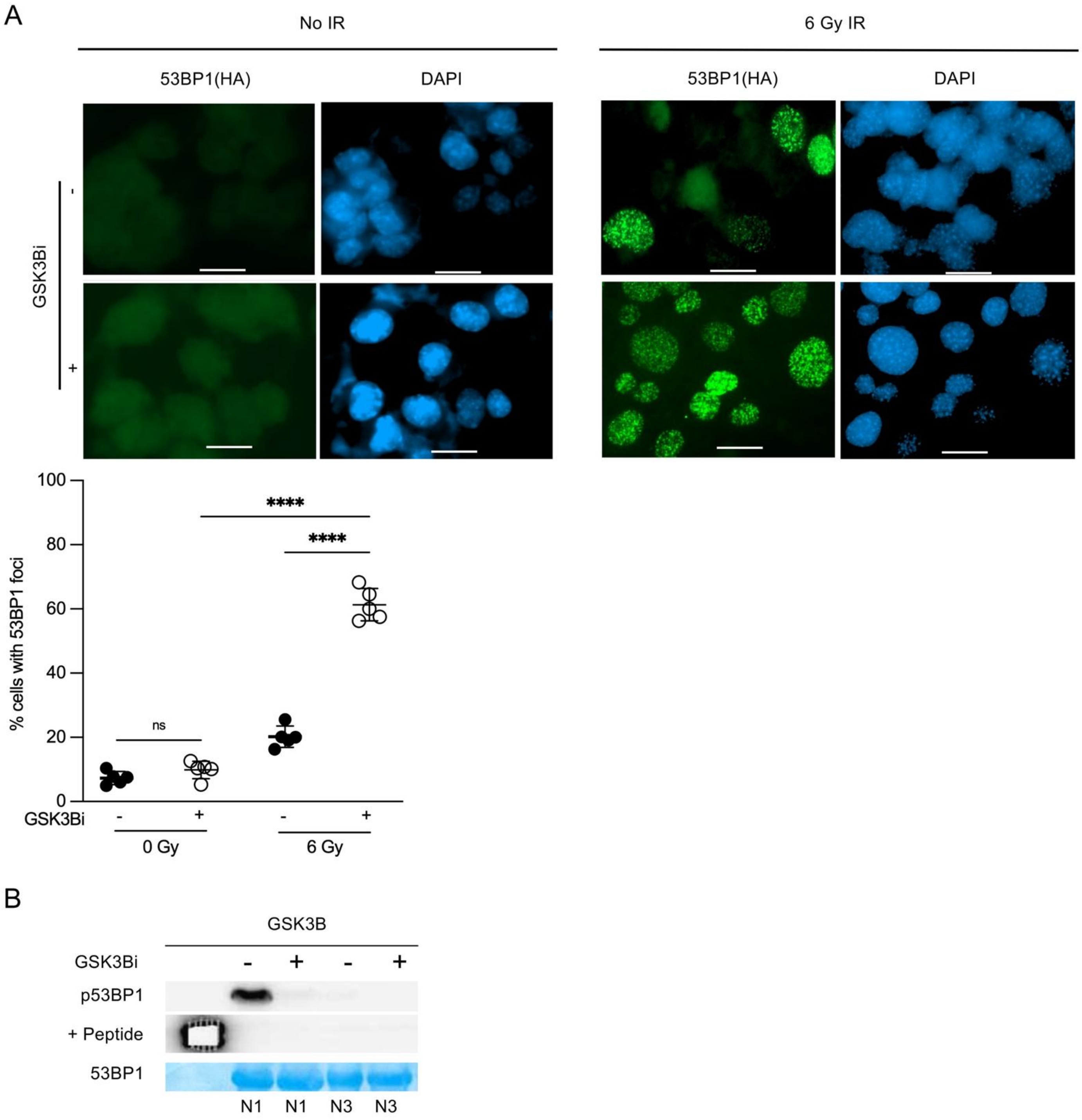

Supplemental Figure 1. GSK3B phosphorylates 53BP1 at Threonine 334 in HN33 cells

(A) Representative images and quantification of 53BP1 foci-stained HN33 cells treated with GSK3Bi (30 um lithium) before and after IR with 6 Gy (n=5). Scale bars represent 100 um. (B) In vitro kinase assay for phosphorylation of 53BP1 by GSK3B.The top row shows the in vitro kinase assay after co-incubating purified GSK3B with 53BP1 fragments N1 (aa 1-361) or N3 (aa 667-1025). The middle row shows the GSK3B-based peptide serving as a positive control. The bottom row shows loading levels of 53BP1 fragments. Values are mean ± SEM. Statistical significance was determined by two-way ANOVA followed by Tukey's multiple comparisons test. ****P < 0.0001.

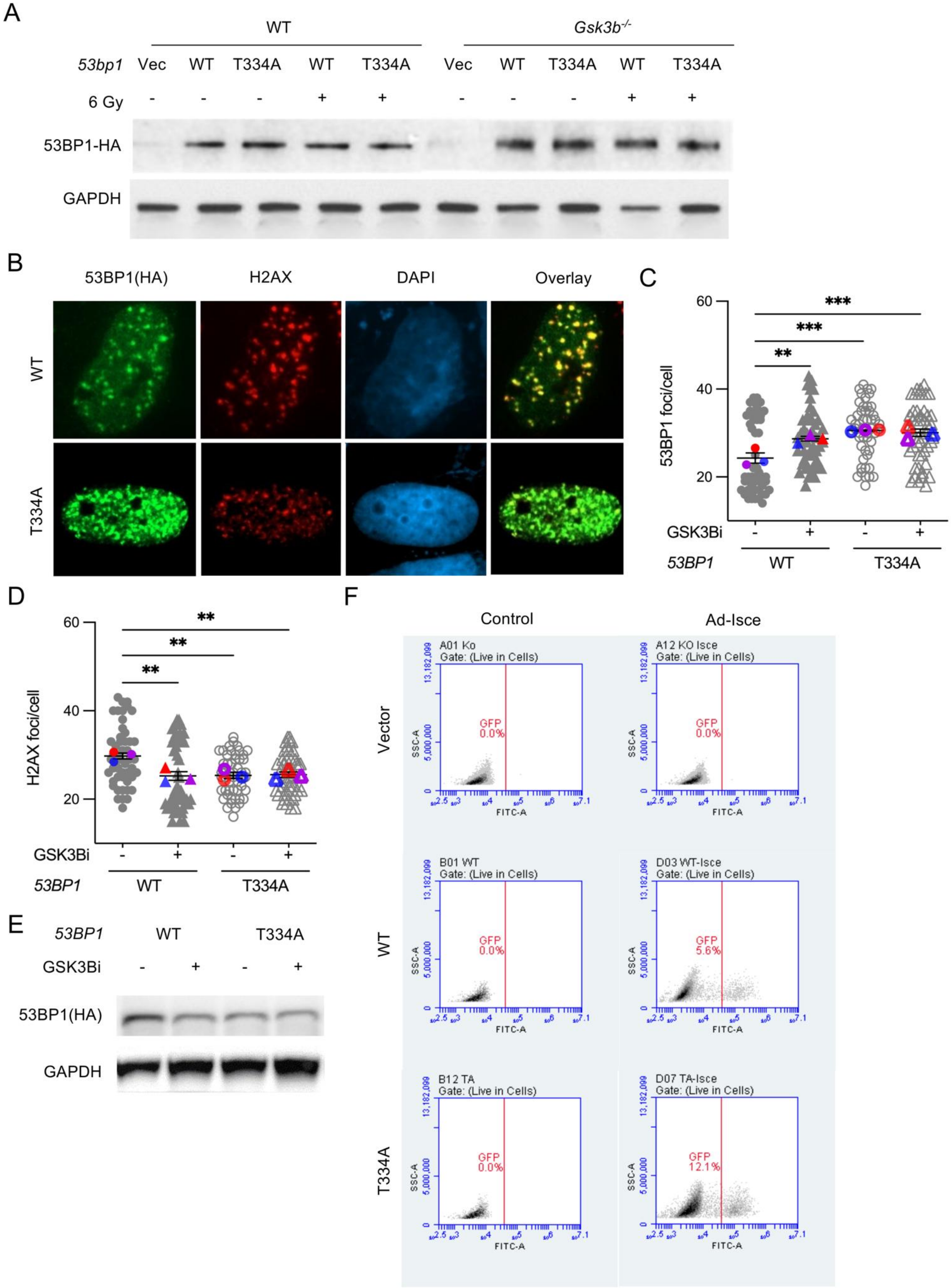

Supplement Figure 2. Effects of GSK3B-mediated phosphorylation at T334 on 53BP1 function in NHEJ

(A) Western blot of 53BP1 protein expression in *Gsk3b*$^{+/+}$ and *Gsk3b*$^{+/+}$ MEF cells with and without exposure to 6 Gy IR. (B) Representative images of 53BP1 and γH2AX foci in U2OS cells reconstituted with WT or T334A 53BP1. (C) 53BP1 foci per U2OS cell with and without GSK3B inhibitor (n=3). (D) H2AX foci per U2OS cell with and without GSK3B inhibitor (n=3). (E) Western blot of HA-tagged WT and T334A *53BP1* U2OS cells with and without GSK3B inhibitor. (F) Representative FACS profiles of the U2OS PEJ2 cell lines for NHEJ assay. The number of green (GFP+) cells was determined by FACS analysis, and typical FACS traces are shown. Statistical significance was assessed using two-way ANOVA with Tukey's test. **P < 0.01; ****P* < 0.001; ****P < 0.0001

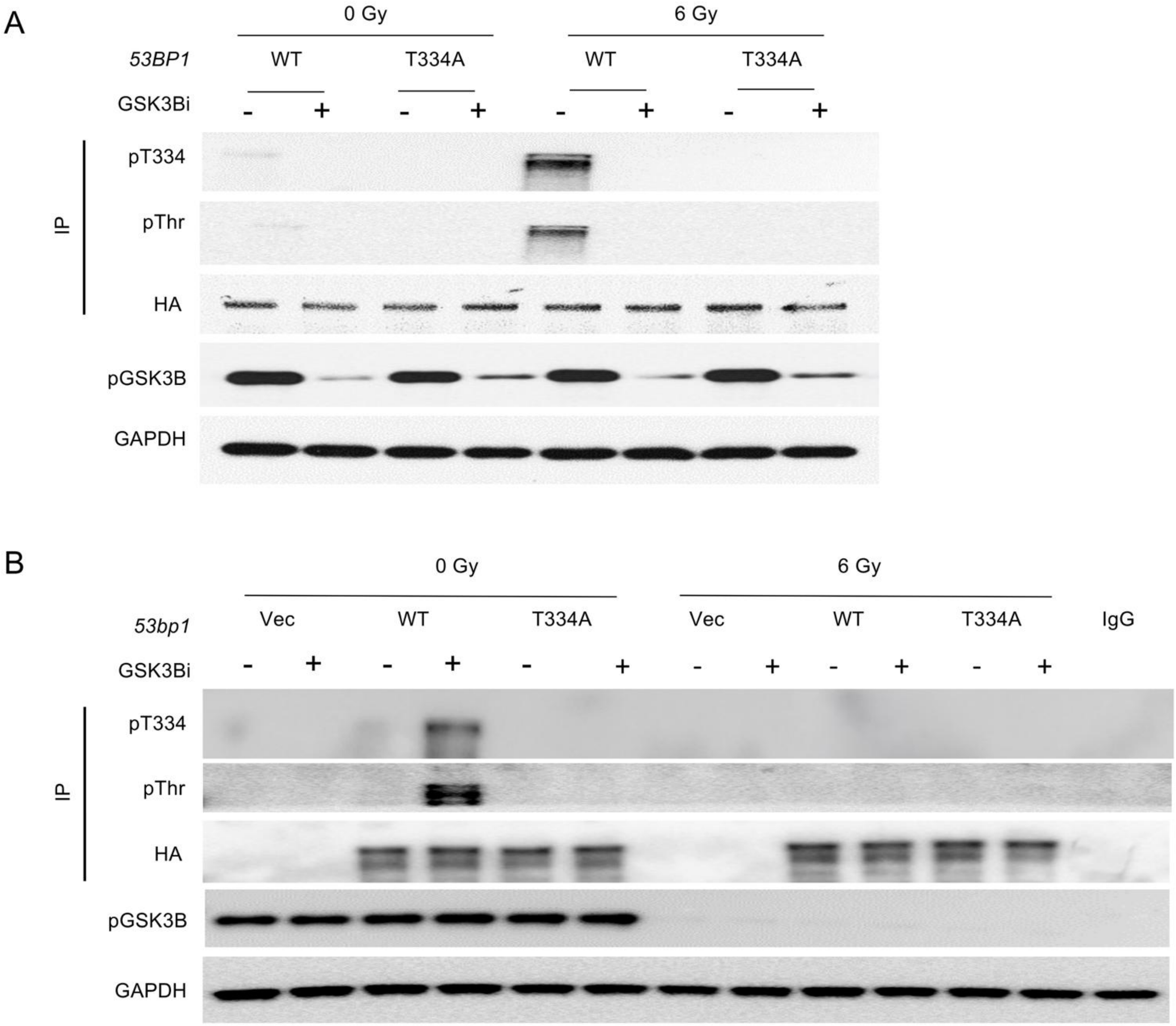

Supplemental Figure 3. Phosphorylation of 53BP1 at threonine 334 modulates its recruitment kinetics to DNA damage sites following ionizing radiation

53BP1 was pulled down with 53BP1 antibody and probed with T334 phospho-specific antibody and pThr (A) in U2OS-KO cells or (B) MEF WT or *GSK3B*$^{-/-}$ cells transfected with WT or T334A *53BP1* before and after IR 6 Gy.

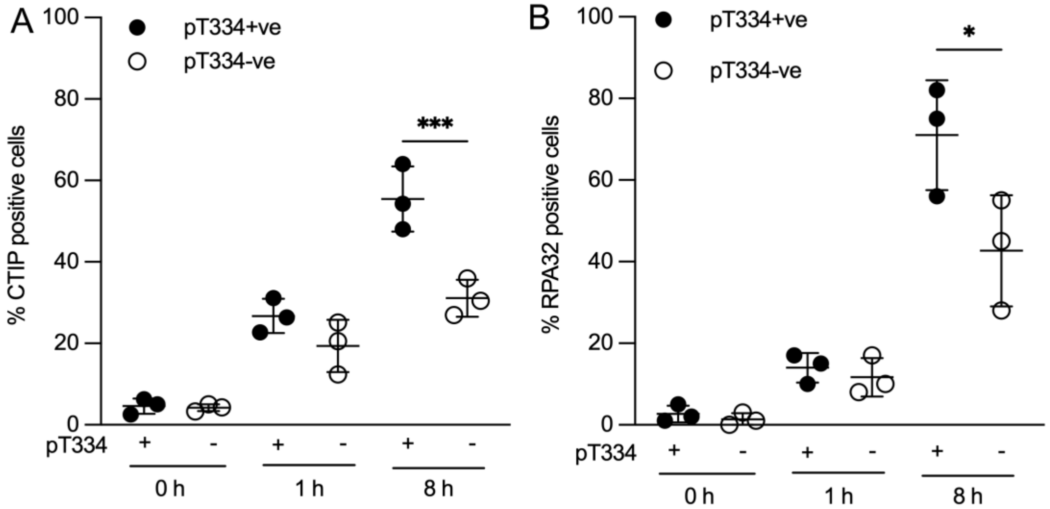

Supplemental Figure 4. The role of T334 phosphorylation on homologous recombination repair.

(A) Percentage of *53BP1*$^{-/-}$ U2OS cells staining for CTIP stratified by pT334A status at different time points following radiation. n=3. (B) Percentage of *53BP1*$^{-/-}$ U2OS cells staining for pRPA32 stratified by pT334A status at different time points following radiation. n=3. Values are mean ± SEM. Statistical significance was assessed using one-way ANOVA followed by Tukey's multiple comparison test. *$P < 0.05$; ***$P < 0.001$.

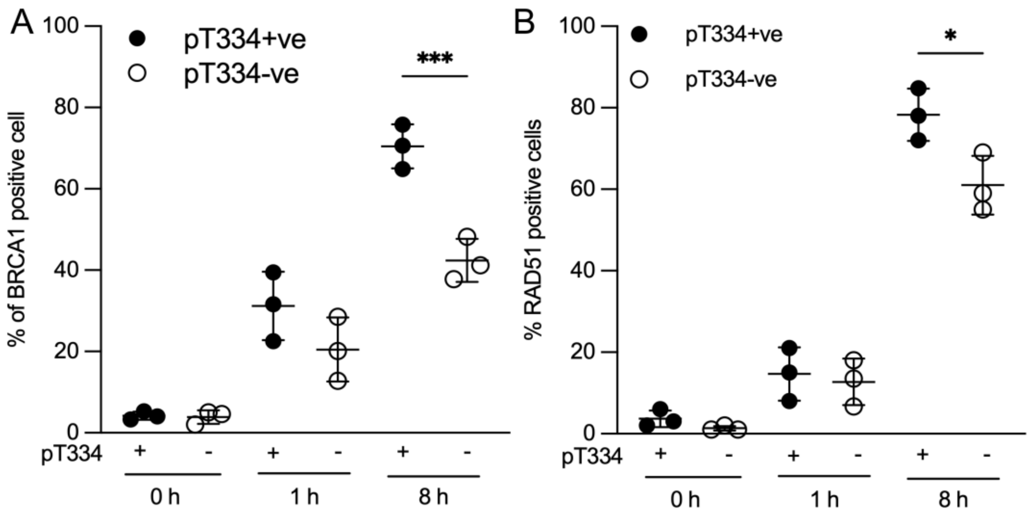

Supplemental Figure 5. The role of T334 phosphorylation on homologous recombination repair.

(A) Percentage of *53BP1*$^{-/-}$ U2OS cells staining for BRCA1 stratified by pT334A status at different time points following radiation. n=3. (B) Percentage of *53BP1*$^{-/-}$ U2OS cells staining for RAD51 stratified by pT334A status at different time points following radiation. n=3. Values are mean ± SEM. Statistical significance was assessed using one-way ANOVA followed by Tukey's multiple comparison test. $^{*}P < 0.05$; $^{***}P < 0.001$.

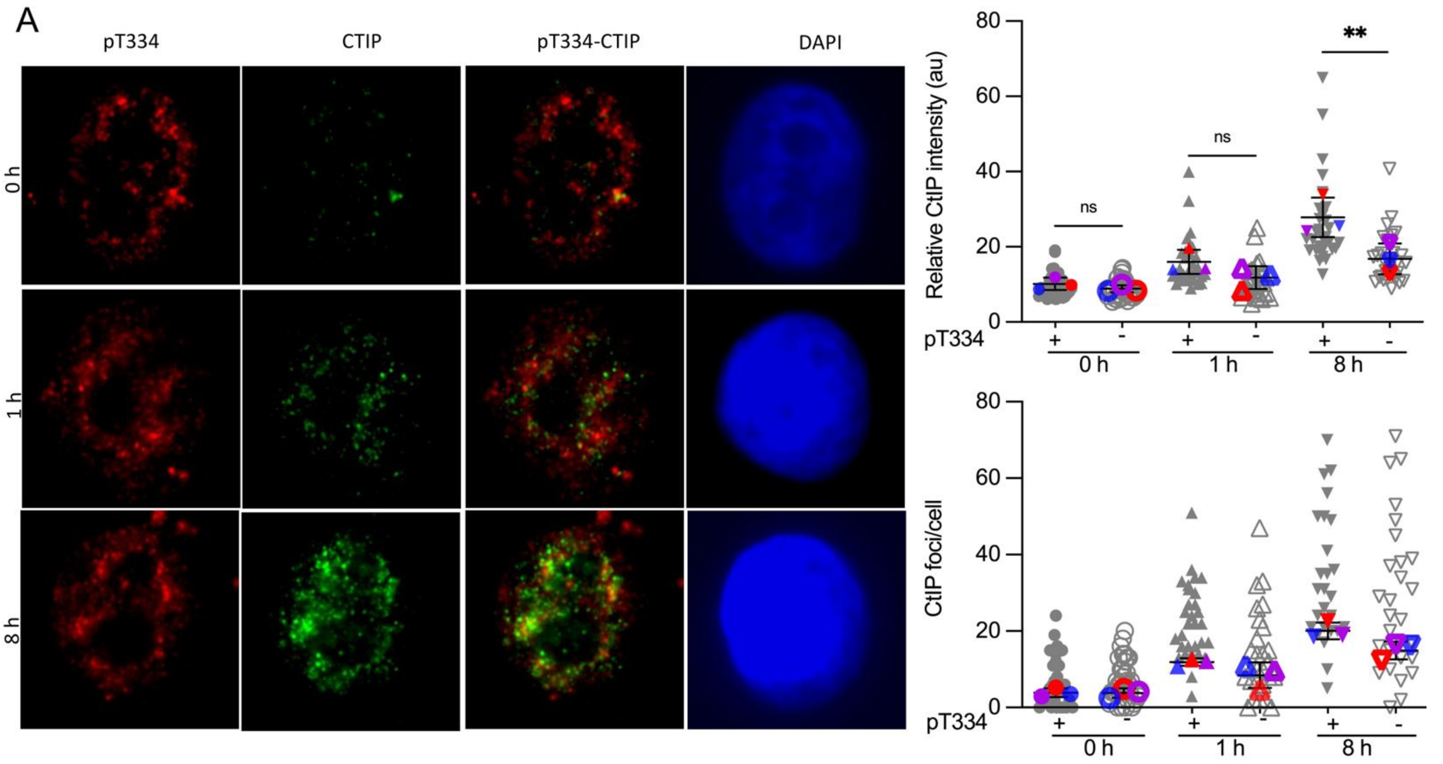

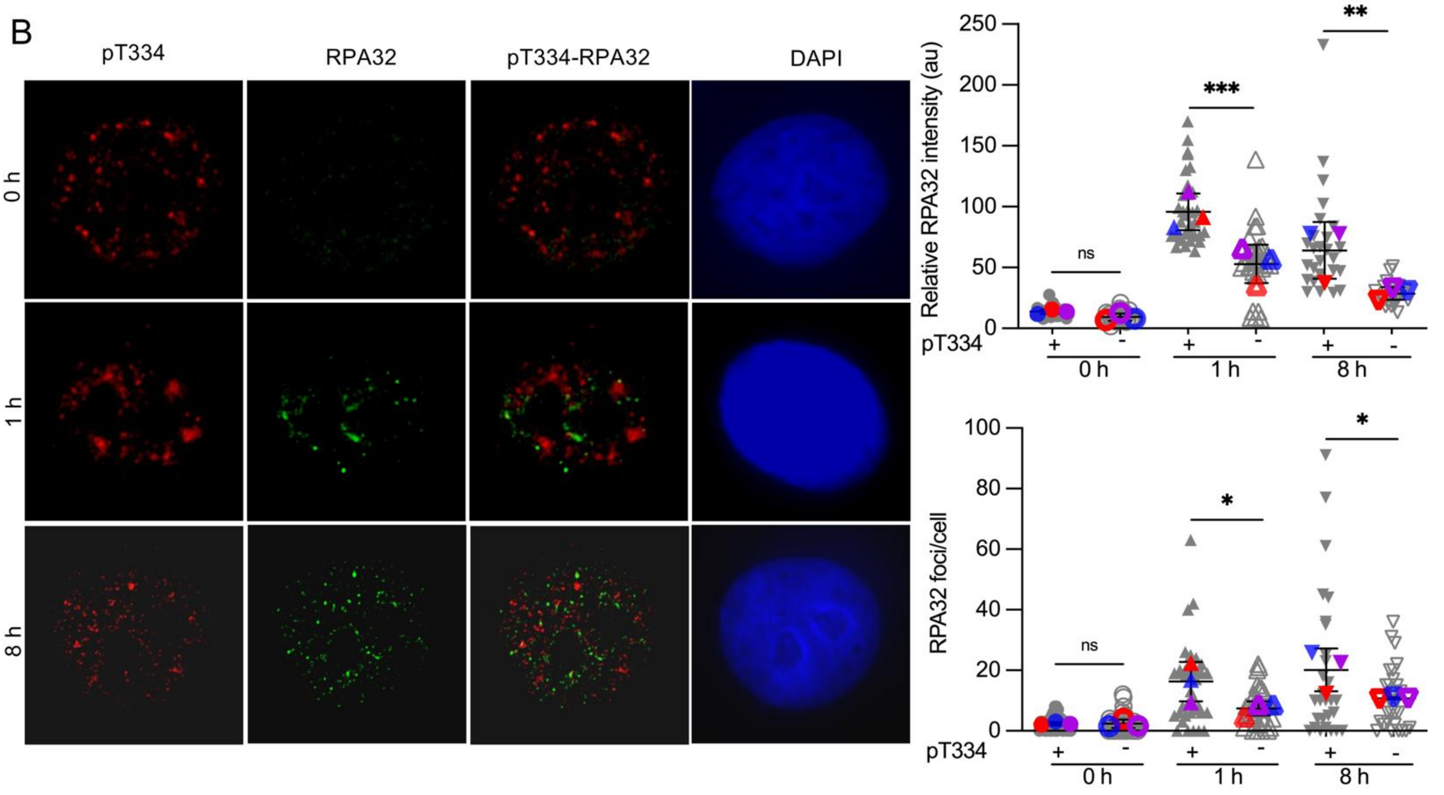

Supplemental Figure 6. The role of T334 phosphorylation on homologous recombination repair.

(A) Representative images of MCF7 cells irradiated with 6 Gy and co-stained for CtIP and p- T334 phospho-specific antibodies. Number of CtIP foci per cell stratified by pT334 status at different time points following radiation and relative intensity of CtIP foci at various time points. n=3. (B) Representative images of MCF7 cells irradiated with 6 Gy and co-stained for pRPA32 and pT334 phospho-specific antibodies. Number of pRPA32 foci per cell stratified by pT334 status at different time points following radiation and relative intensity

of pRPA32 foci at various time points. n=3. Values are mean ± SEM. Statistical significance was assessed using one-way ANOVA followed by Tukey's multiple comparison test. ****P < 0.0001.

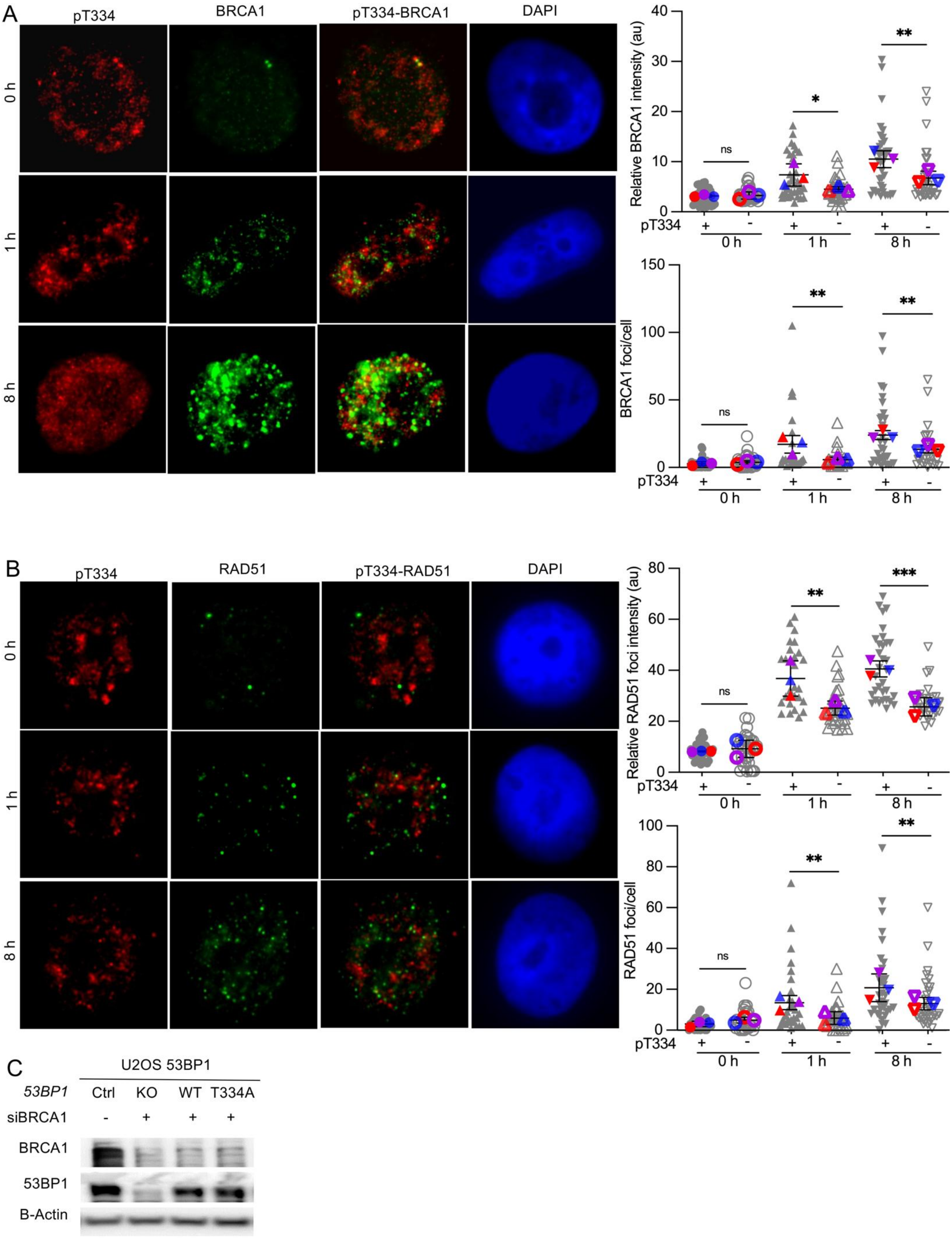
A
pT334
BRCA1
pT334-BRCA1
DAPI
0 h
1 h
8 h
Relative BRCA1 intensity (au)
BRCA1 foci/cell
ns
*
**
pT334
+
-
0 h
1 h
8 h
B
pT334
RAD51
pT334-RAD51
DAPI
Relative RAD51 foci intensity (au)
RAD51 foci/cell
***
C
U2OS 53BP1
53BP1
Ctrl
KO
WT
T334A
siBRCA1
BRCA1
53BP1
B-Actin

Supplemental Figure 7. The role of T334 phosphorylation on homologous recombination repair.

(A) Representative images of MCF7 cells irradiated with 6 Gy and co-stained for BRCA1 and T334 phospho-specific antibodies. Number of BRCA1 foci per cell stratified by pT334 status at different time points following radiation and relative intensity of BRCA1 foci at various time points. n=3. (B) Representative images of MCF7 cells irradiated with 6 Gy and co-stained for RAD51 and T334 phospho-specific antibodies. Number of RAD51 foci per cell stratified by pT334 status at different time points following radiation and relative intensity of RAD51 foci at various time points. n=3. Quantification of foci and intensity were calculated for each cell by the Image J software. n=3. (C) Western blot of BRCA1 and 53BP1 in U2OS cells with various 53BP1 alleles with and without BRCA1 knockdown. Values are mean ± SEM. Statistical significance was assessed using one-way ANOVA followed by Tukey's multiple comparison test and one-way ANOVA with Bonferroni’s correction. $^{**}P < 0.01$; $^{****}P < 0.0001$.

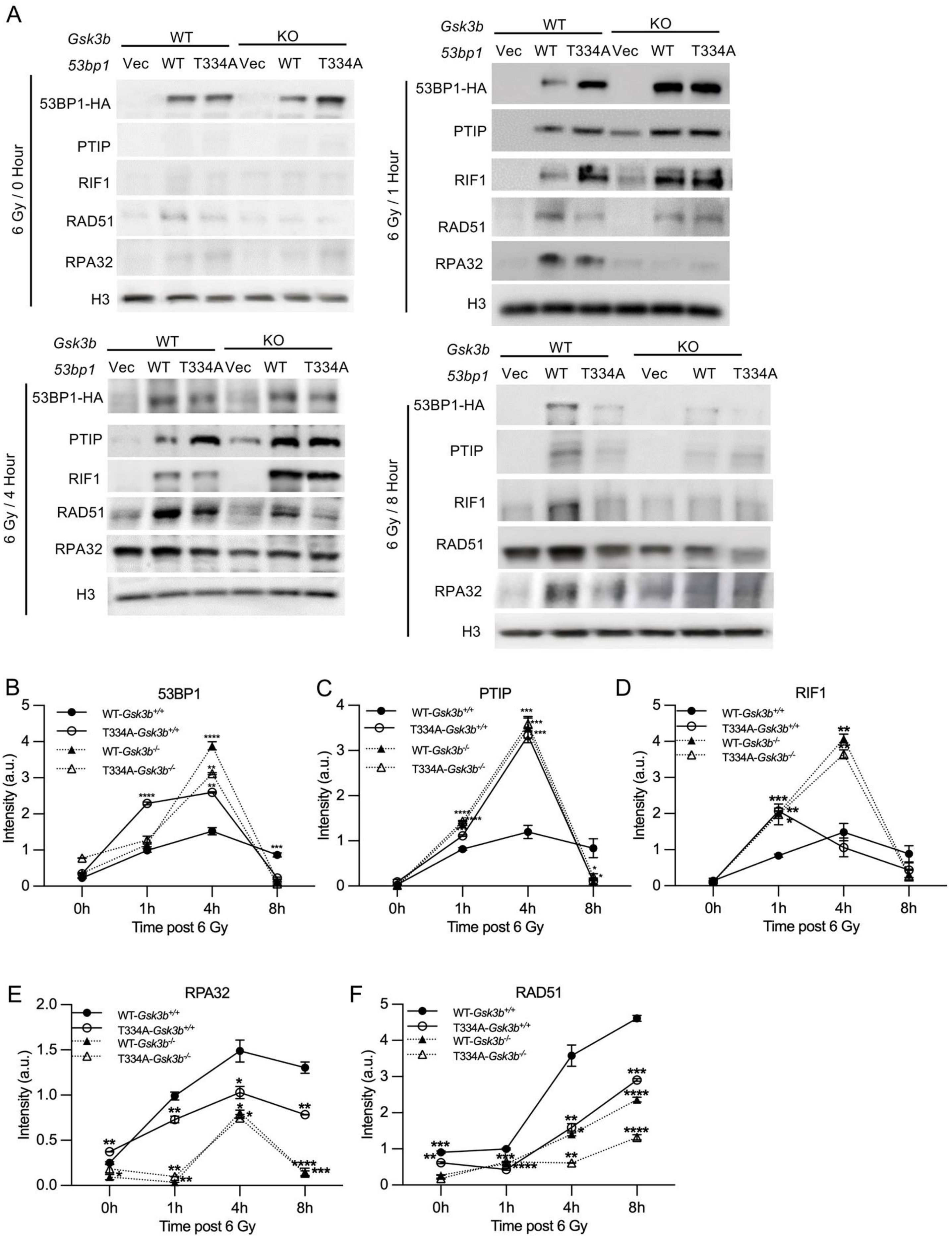
A
Gsk3b
WT
KO
53bp1
Vec
WT
T334A
53BP1-HA
PTIP
RIF1
RAD51
RPA32
H3
6 Gy / 0 Hour
6 Gy / 1 Hour
6 Gy / 4 Hour
6 Gy / 8 Hour
B
53BP1
C
PTIP
D
RIF1
E
RPA32
F
RAD51
WT-Gsk3b+/+
T334A-Gsk3b+/+
WT-Gsk3b-/-
T334A-Gsk3b-/-
Intensity (a.u.)
Time post 6 Gy
0h
1h
4h
8h

Supplemental Figure 8. T334 phosphorylation modulates 53BP1 interaction with PTIP and RIF1 and influences DSB repair dynamics

(A) Chromatin binding assay in WT and *GSK3B*$^{-/-}$ MEF cells with various 53BP1 alleles and GSK3B status after irradiation with 6 Gy. Quantification of chromatin binding with (B) 53BP1(HA), (C) PTIP, (D) RIF1, (E) RPA32, and (F) RAD51 at 0, 1, 4, and 8 hours following IR. n=3. Values are mean ± SEM. Statistical significance was determined by two-way ANOVA followed by Tukey’s multiple comparisons test. *P < 0.05; **P < 0.01; ***P < 0.001; ****P < 0.0001.

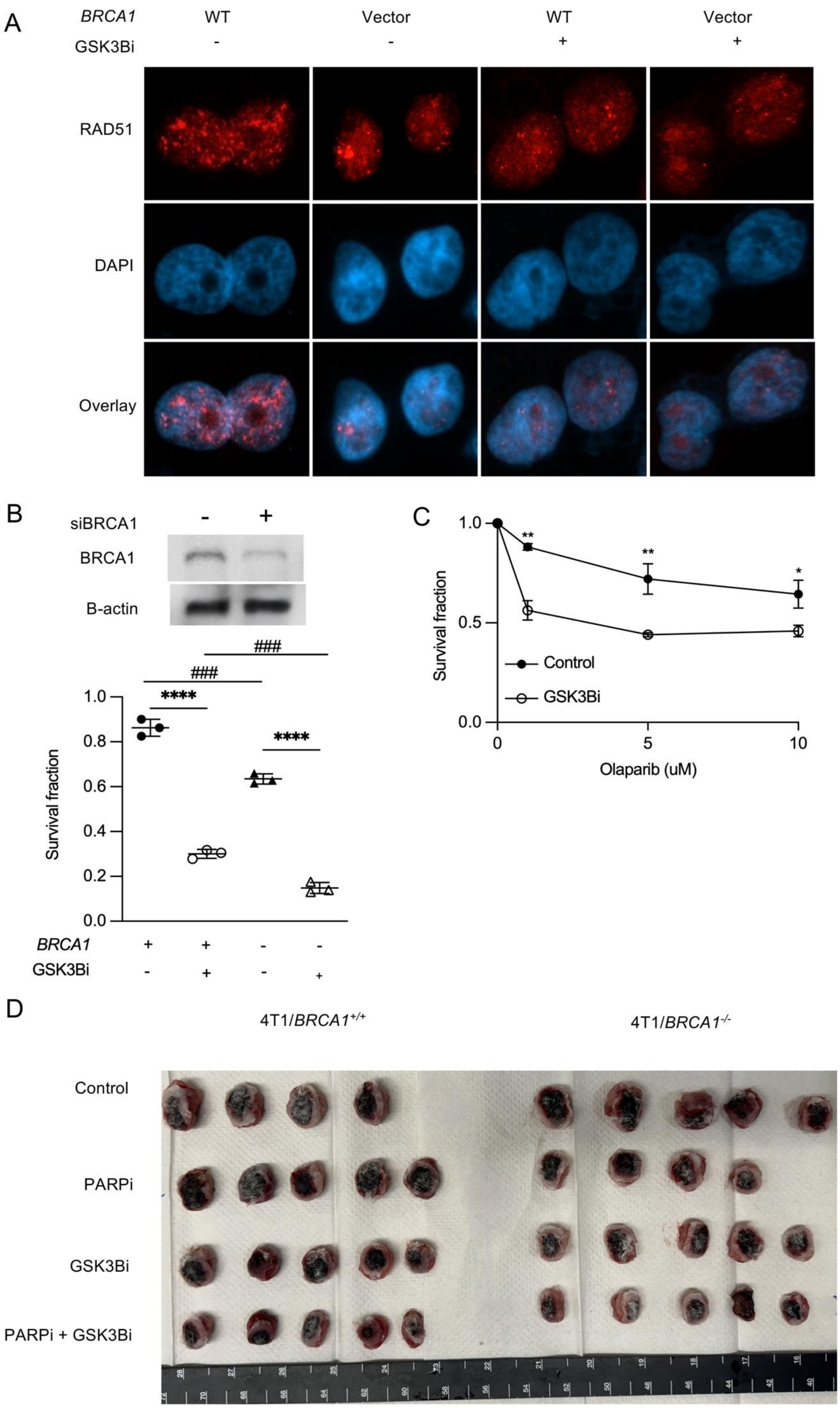
A
BRCA1
WT
Vector
WT
Vector
GSK3Bi
-
-
+
+
RAD51
DAPI
Overlay
B
siBRCA1
-
+
BRCA1
B-actin
###
###
****
****
Survival fraction
1.0
0.8
0.6
0.4
0.2
0.0
BRCA1
+
+
-
-
GSK3Bi
-
+
-
+
C
1.0
0.5
0.0
**
**
*
Survival fraction
Control
GSK3Bi
0
5
10
Olaparib (uM)
D
4T1/BRCA1+/+
4T1/BRCA1-/-
Control
PARPi
GSK3Bi
PARPi + GSK3Bi

Supplemental Figure 9. T334 phosphorylation of 53BP1 is critical for chromosomal HR efficiency and determines synthetic lethality of PARPi independent BRCA1 status

(A) Representative images of *BRCA1*-deficient MDA-MB436 cells with or without BRCA1 reconstituted; with or without GSK3B inhibitor incubated with 15 uM of olaparib. Cells were stained for RAD51. (B) Upper panel shows western blot of BRCA1 expression in 4T1 cells with and without *BRCA1* knockdown. Lower panel is relative survival fraction of 4T1 cells with or without *BRCA1* knockdown and with or without GSK3B inhibitor incubated with 15 uM of olaparib. n=3. (C) Survival fractions of MCF-7 cells in the presence or absence of GSK3B inhibitor subjected to the indicated concentrations of Olaparib. n=3. (E) Representative images of 4T1 fat pad tumors. Values are mean ± SEM. Statistical significance was determined by two-way ANOVA followed by Tukey's multiple comparisons test. *$P < 0.05$; **$P < 0.01$; ****$P < 0.0001$; $^{\#\#}P < 0.01$; $^{\#\#\#}P < 0.001$.

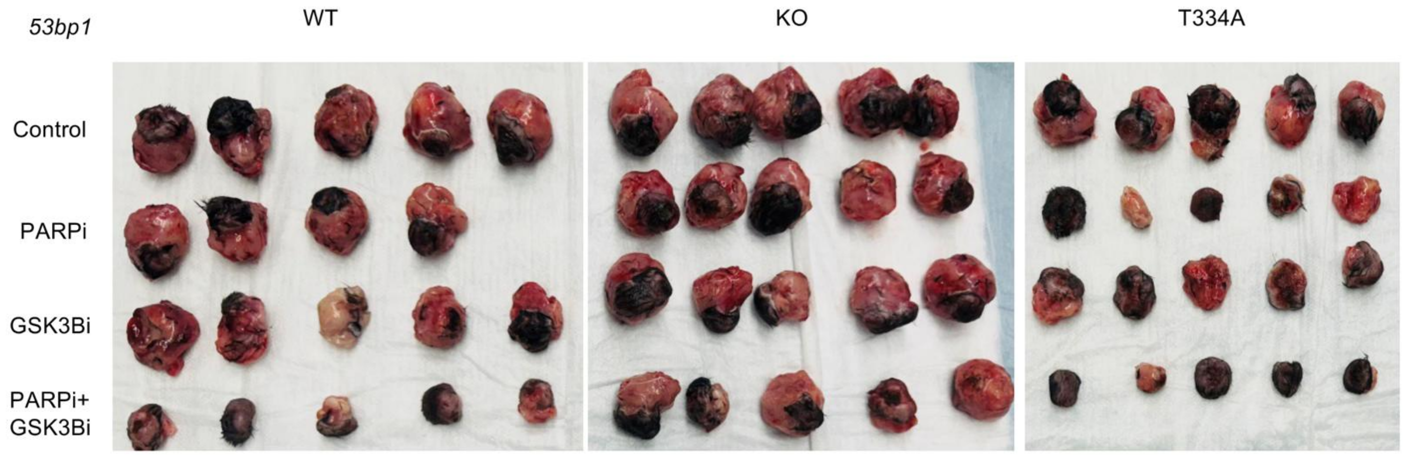

Supplemental Figure 10. GSK3B inhibition-mediated sensitization to PARPi requires functional 53BP1

Representative images of SB28 subcutaneous tumors
