# Supplementary material for: GSK3B directs DNA repair choice and determines tumor response to PARP1 inhibition independent of BRCA1
Source: J Clin Invest. 2025 Nov 17;135(22):e189956. doi: 10.1172/JCI189956 (PMC12618078; doi:10.1172/JCI189956)

Fig 1B

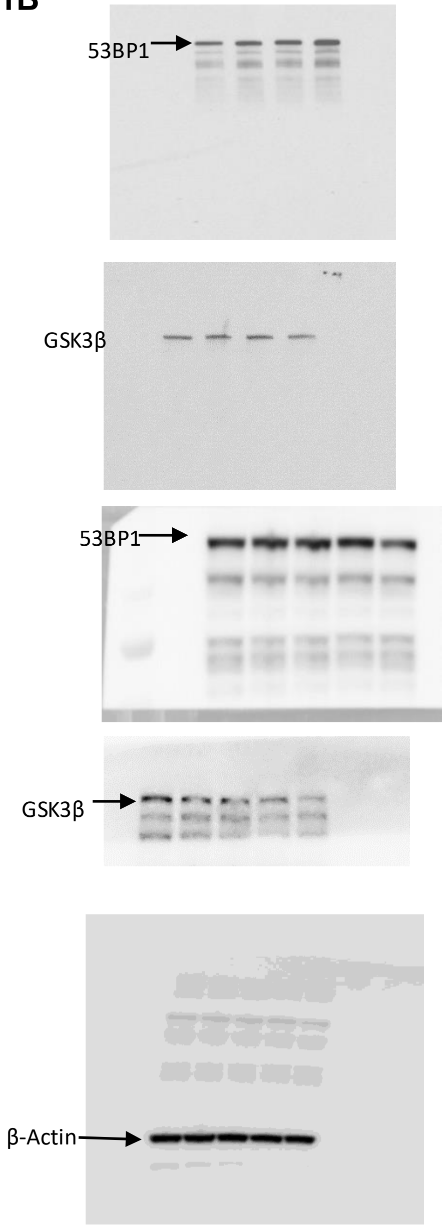

Fig 1D

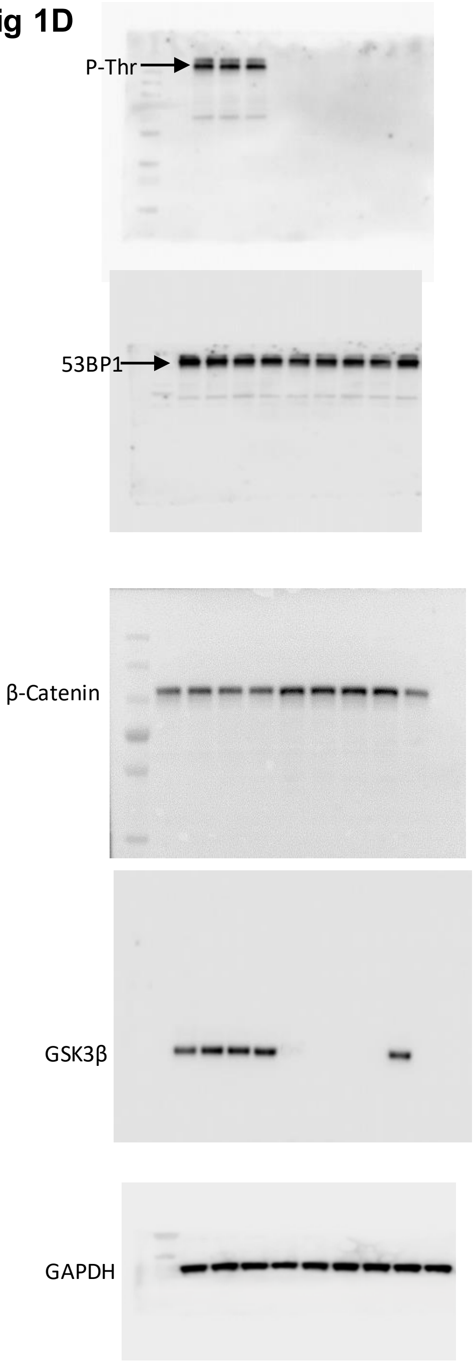

Fig 1E

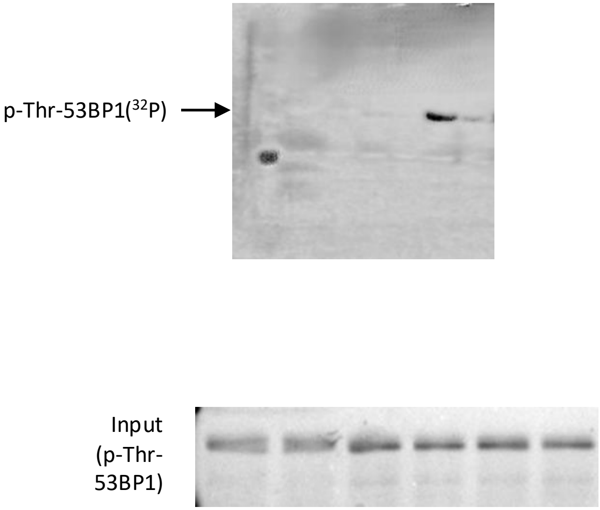

Fig 4F

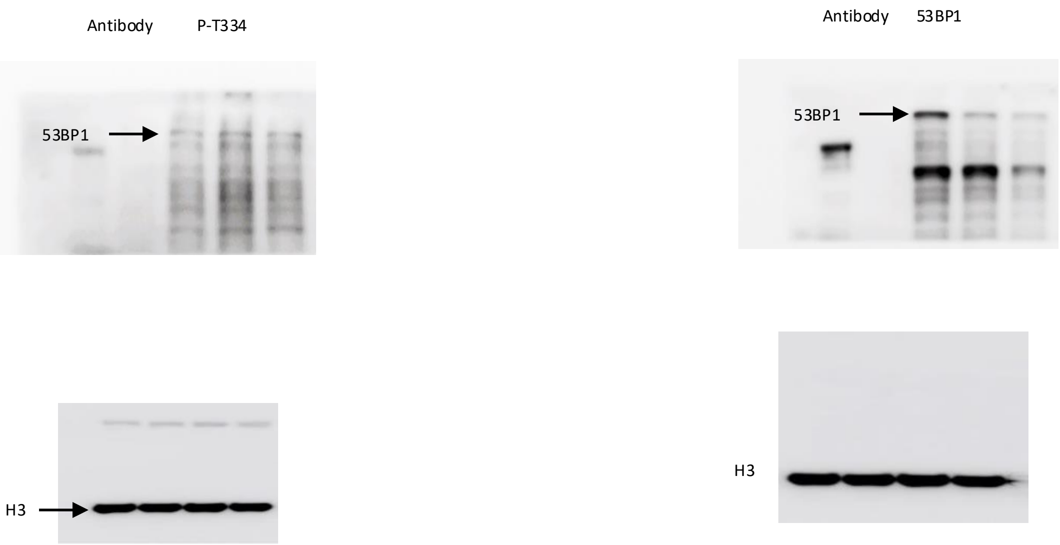

Figure 7A

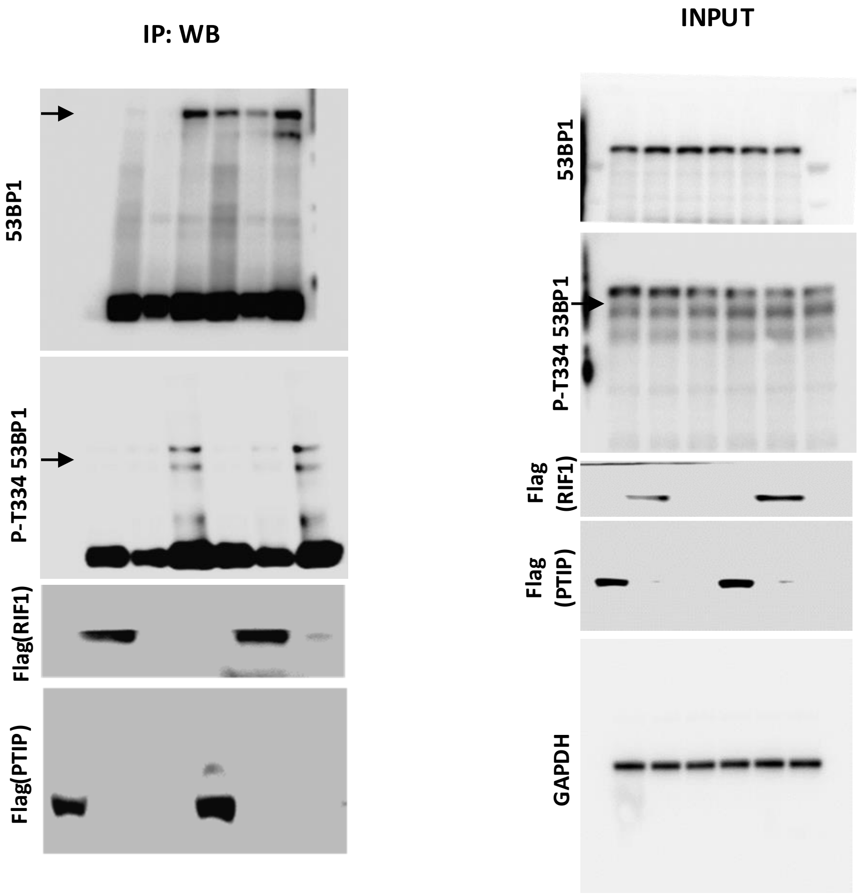

IP: WB
53BP1
P-T334 53BP1
Flag(RIF1)
Flag(PTIP)
INPUT
53BP1
P-T334 53BP1
Flag (RIF1)
Flag (PTIP)
GAPDH

Figure 8

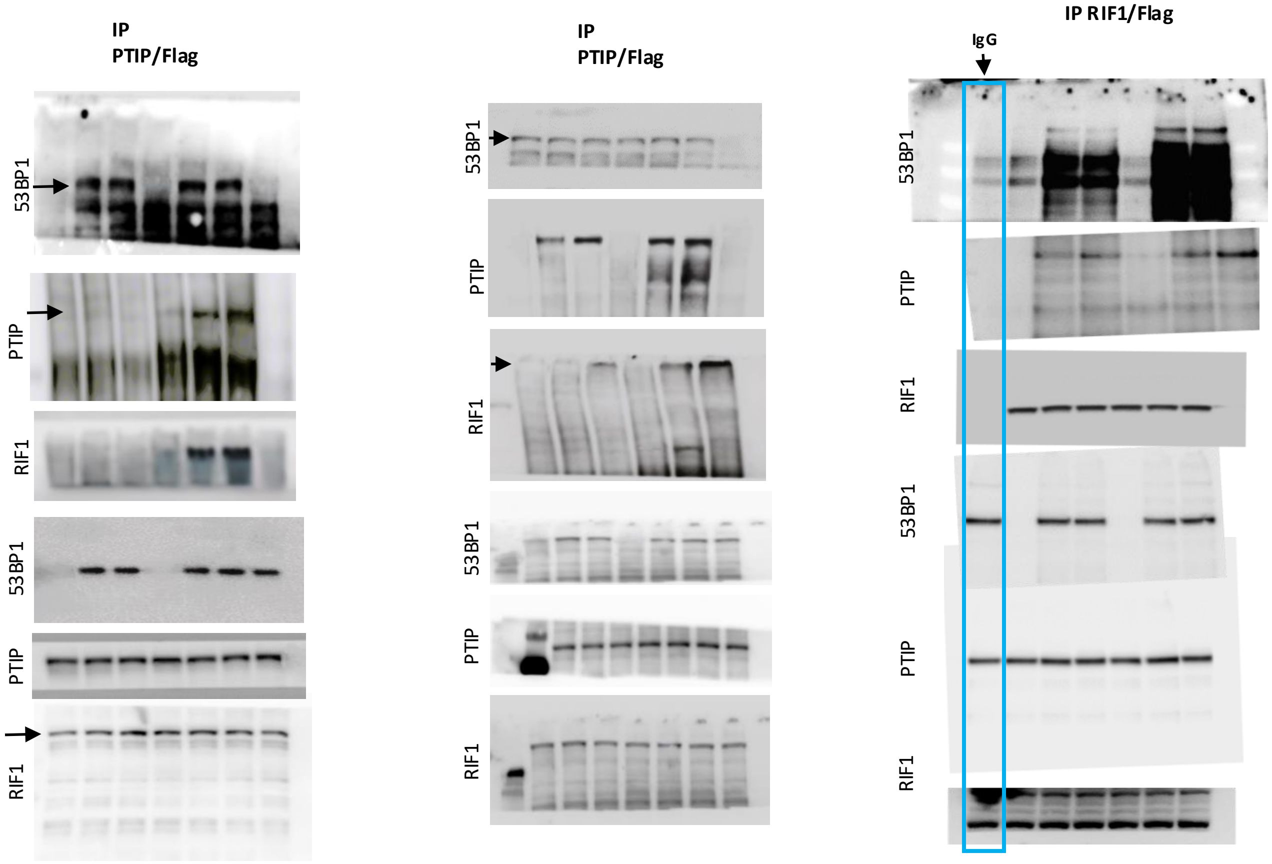
IP
PTIP/Flag
53BP1
PTIP
RIF1
53BP1
PTIP
RIF1
IP
PTIP/Flag
53BP1
PTIP
RIF1
53BP1
PTIP
RIF1
IP RIF1/Flag
IgG
53BP1
PTIP
RIF1
53BP1
PTIP
RIF1

Figure 9A

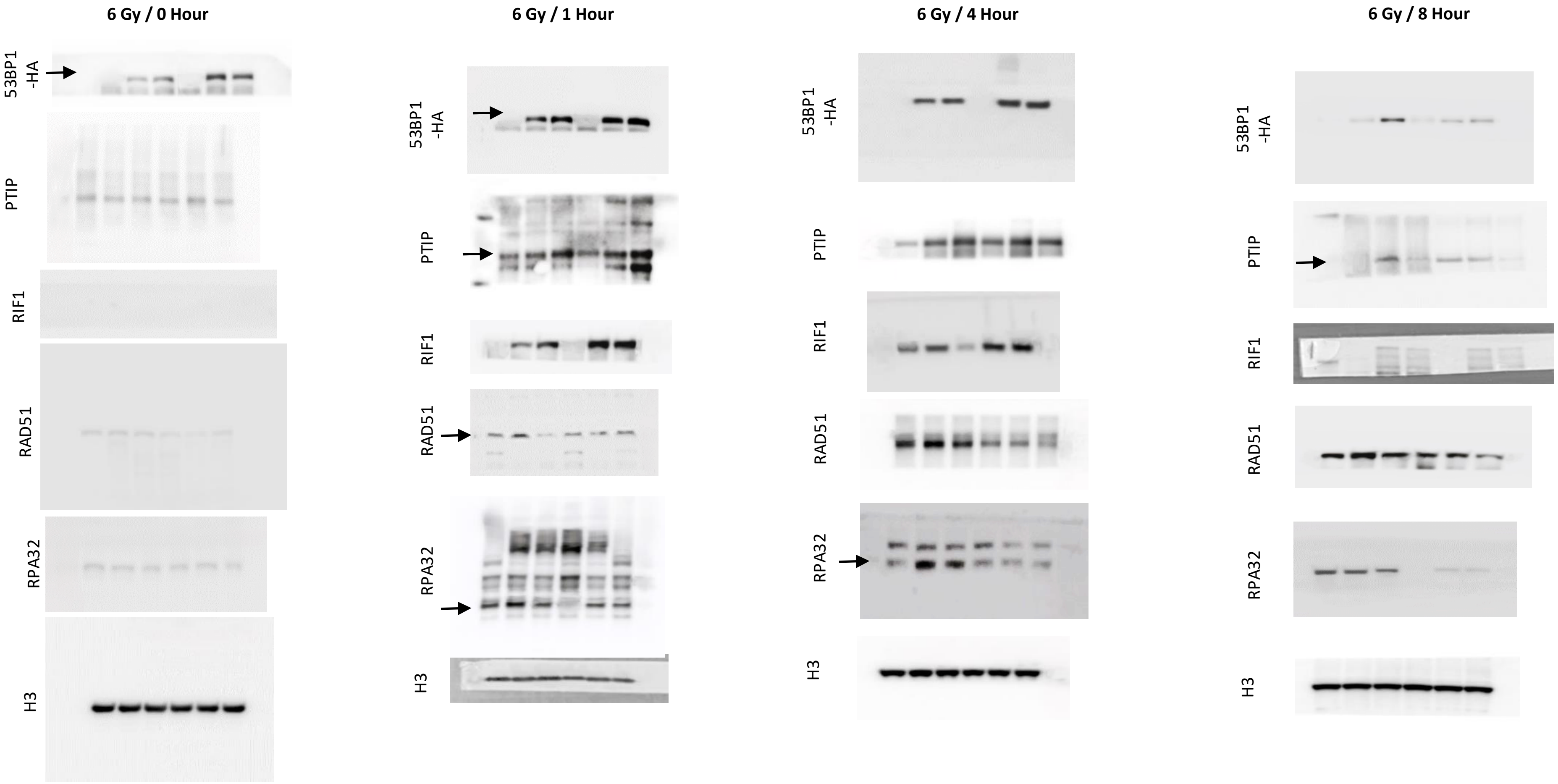
6 Gy / 0 Hour
6 Gy / 1 Hour
6 Gy / 4 Hour
6 Gy / 8 Hour
53BP1 -HA
PTIP
RIF1
RAD51
RPA32
H3

Supplement Figure 1B

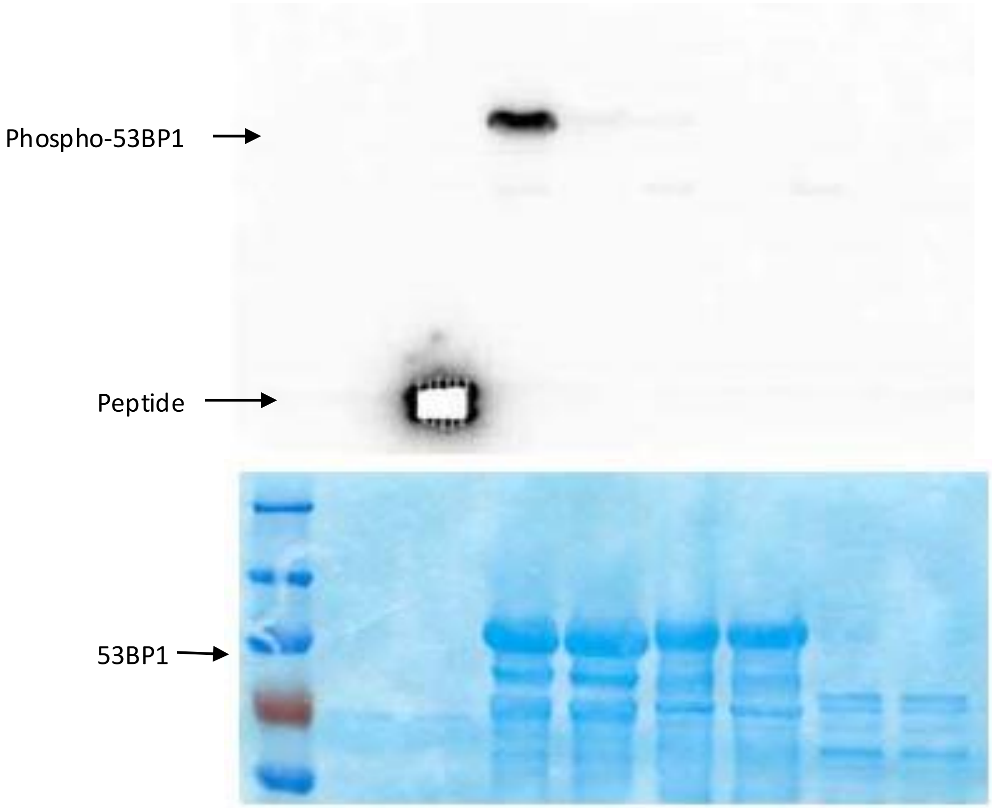

Supplemental Figure 2A

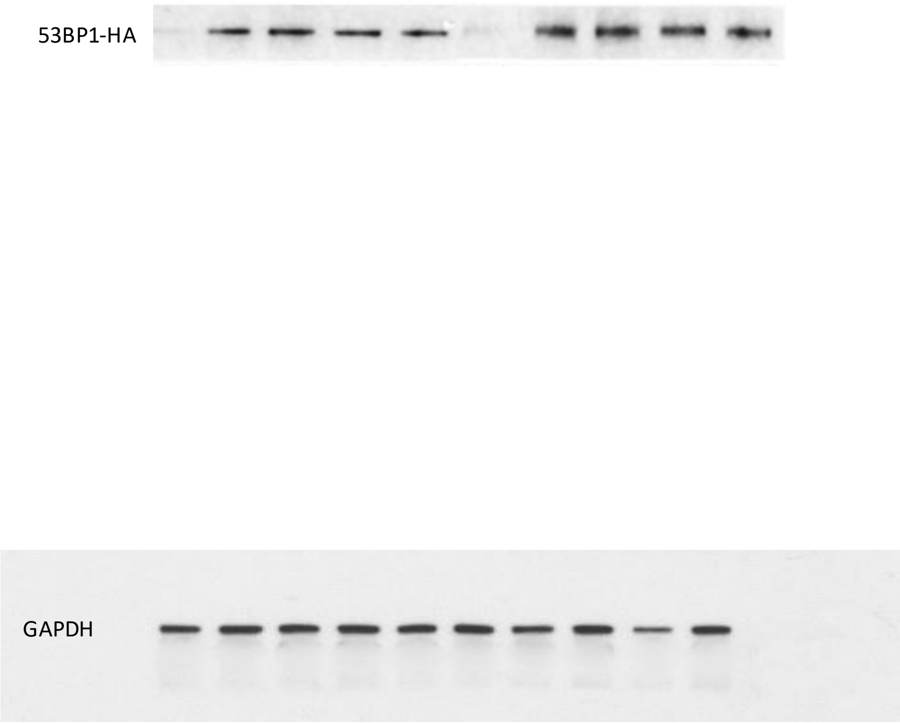

Supplemental Figure 2E

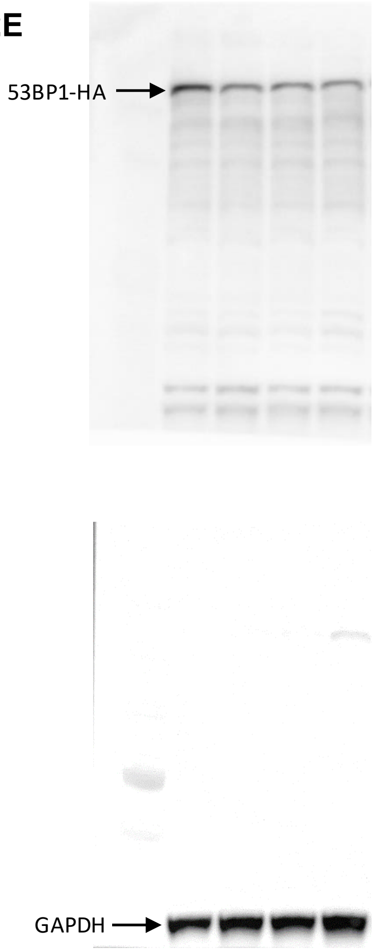

Supplement Figure 4

4A

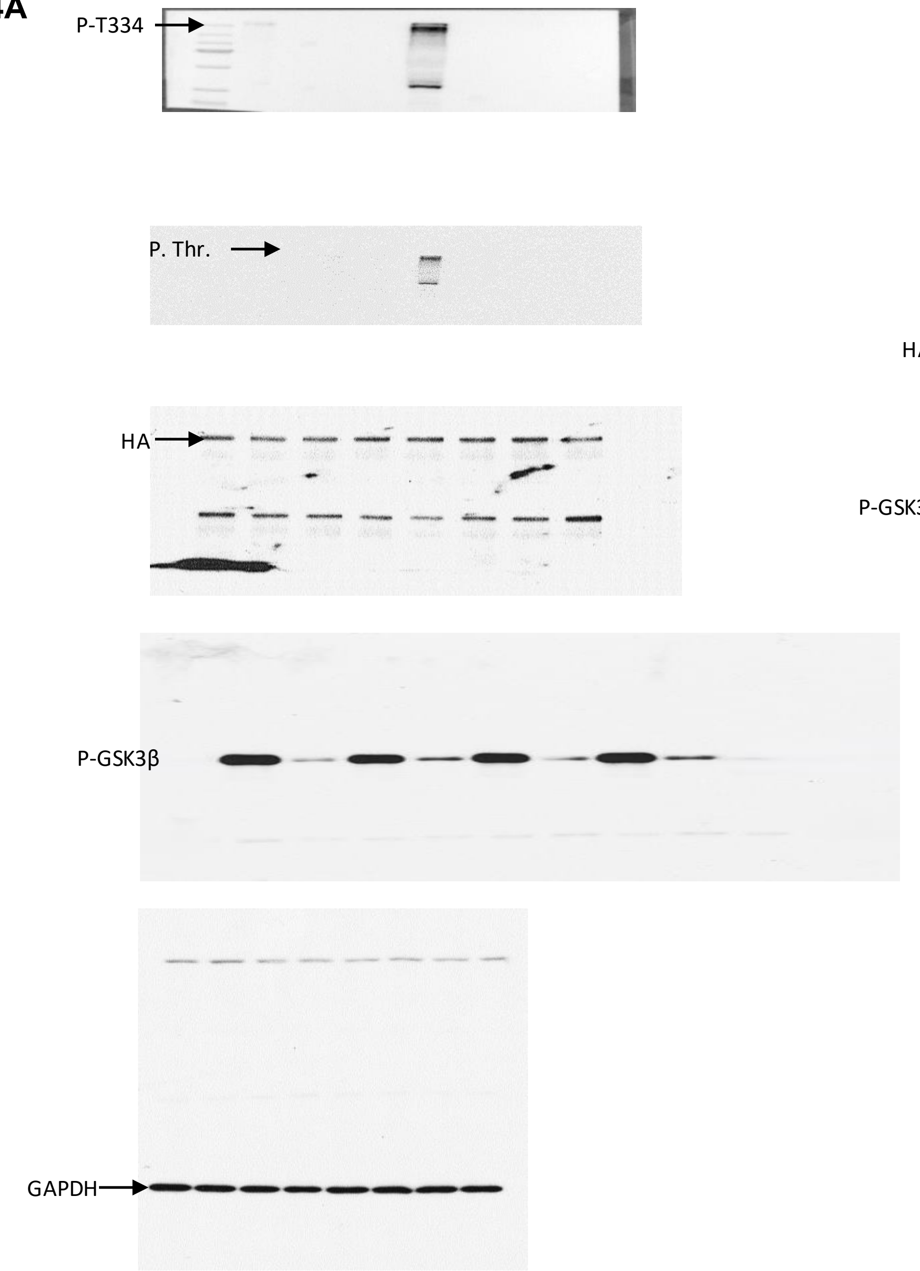

4B

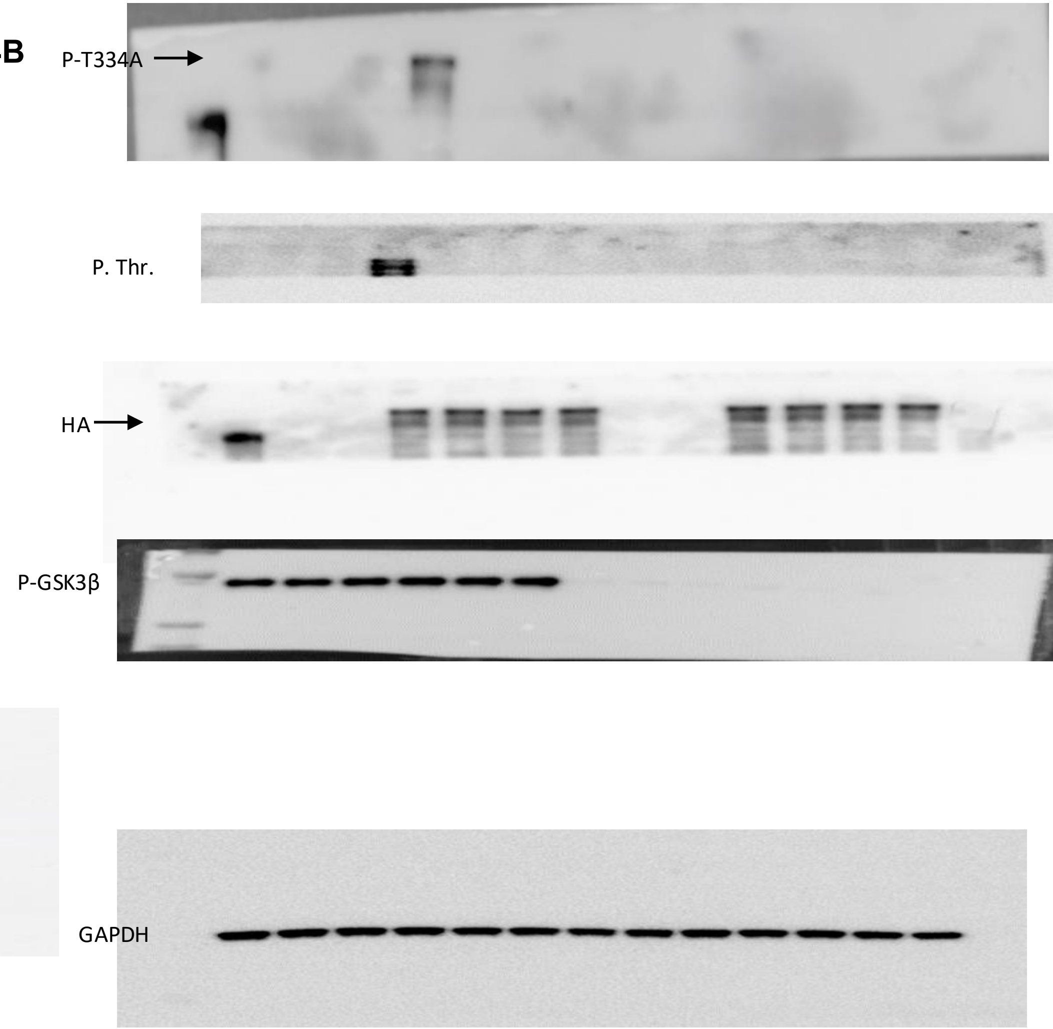

Supplement Figure 8C

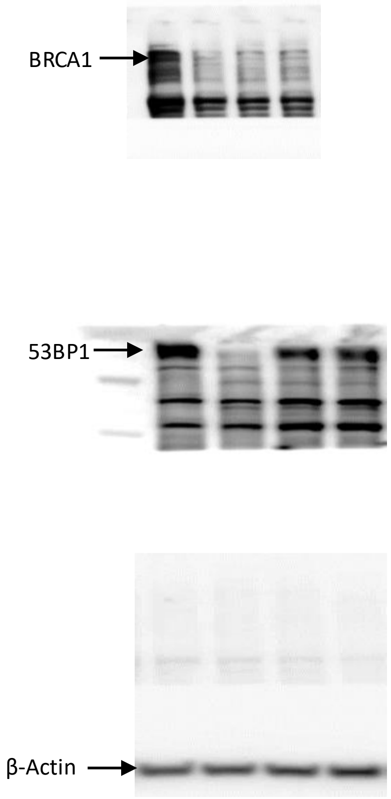

Supplement Figure 9A

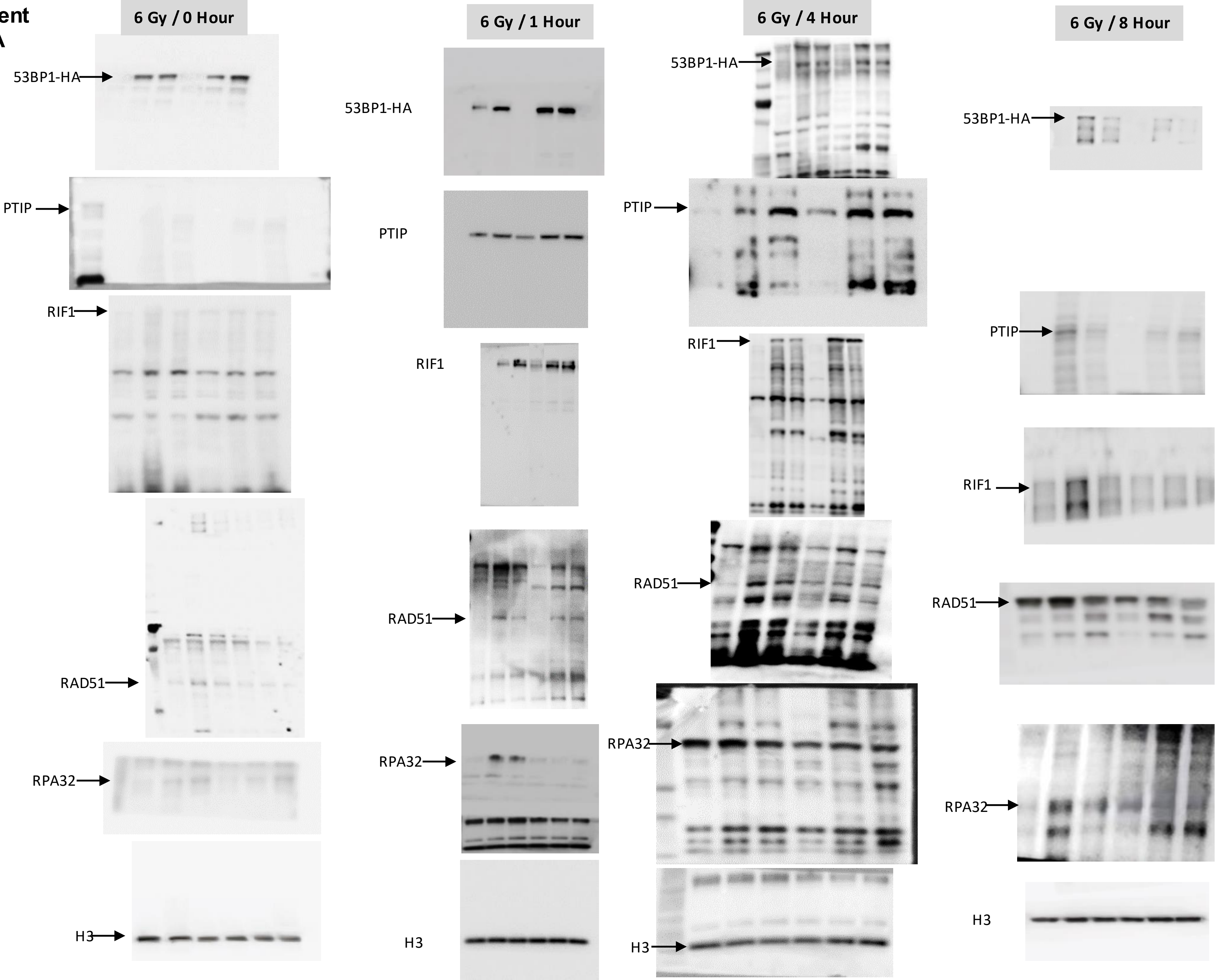

Supplement Figure 10B

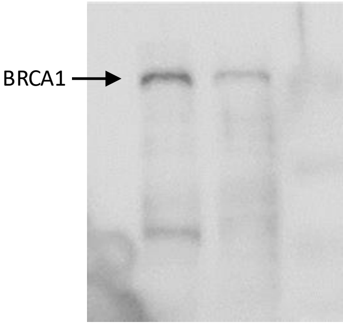

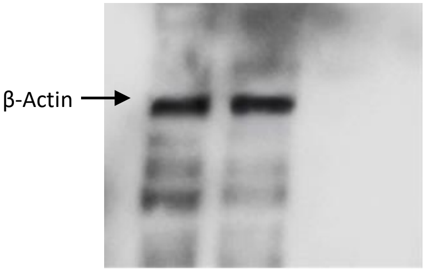

Supplement: Unedited blot and gel images [file jci-135-189956-s133.pdf]
